# Balanophora polysaccharide improves renal injury and fibrosis in db/db diabetic nephropathy mice via NLRP3 inflammasome mediated inflammation

**DOI:** 10.3389/fphar.2025.1671678

**Published:** 2025-11-28

**Authors:** Chaoxi Tian, Aolong Ma, Tianying Song, Fangyu Zhao, Jing Huang, Jianhong Gao, Honglin Yan, Xianbing Chen

**Affiliations:** 1 Hubei Provincial Key Laboratory of Occurrence and Intervention of Rheumatic Diseases, Hubei Minzu University, Enshi, China; 2 Health Science Center, Hubei Minzu University, Enshi, China; 3 Department of Pathology, Renmin Hospital of Wuhan University, Wuhan, China

**Keywords:** balanophora polysaccharide, diabetic nephropathy, renal fibrosis, apoptosis, inflammation, extracellular matrix, NLRP3 inflammasome

## Abstract

**Introduction:**

Diabetic nephropathy (DN) is a major complication of diabetes, with renal fibrosis leading to progressive renal function decline. Understanding interventions for renal injury and fibrosis in DN is vital, and given its complex pathogenesis, new therapeutic agents are urgently needed.

**Methods:**

The DN model was established using db/db mice, which received balanophora polysaccharide (BPS) treatment. The therapeutic efficacy of BPS for DN was evaluated by measuring body weight, fasting blood glucose (FBG), lipid profiles, renal function parameters, serum inflammatory factors, and histopathological changes. Furthermore, the underlying mechanisms by which BPS exerted its therapeutic effects were investigated using transmission electron microscopy (TEM), immunohistochemistry (IHC), immunofluorescence (IF), and Western blotting.

**Results:**

BPS significantly reduced body weight, as well as fasting blood glucose (FBG) and lipid levels in db/db mice. Additionally, it improved renal function and effectively alleviated renal injury. Moreover, BPS decreased the expression of extracellular matrix (ECM) proteins and inhibited ECM deposition, thereby alleviating the progression of renal fibrosis in DN and reducing cell apoptosis. Notably, BPS effectively inhibited the activity of NLRP3 inflammasome in the renal tissue of db/db mice, which in turn mitigated renal inflammatory response and fibrosis.

**Conclusion:**

BPS can improve renal injury and renal fibrosis in db/db diabetic nephropathy mice, which may be related to the decrease of apoptosis, inhibition of inflammation, reduction of ECM, and regulation of NLRP3 inflammasome. This study provides a scientific basis for the clinical application of BPS in the treatment of renal fibrosis in DN and is expected to promote the drug development and clinical application of BPS.

## 1 Introduction

Diabetic nephropathy (DN) is the most common chronic complication of diabetes and also a major cause of end-stage renal disease (ESRD), accounting for 50% of all cases of ESRD (Xiang et al., 2020; Samsu, 2021; Dwivedi and Sikarwar, 2025). The prevalence of diabetes in China has been showing a rapid upward trend year by year, and the latest epidemiological studies have reported that 32.36% of diabetic patients have DN (Jia et al., 2025). The kidney, a vital organ responsible for filtering waste products and maintaining fluid and electrolyte balance in the body, is one of the prime target organs that are acutely vulnerable to the detrimental effects of diabetes, and approximately 20%–40% of diabetic patients will eventually develop DN (Ma, 2018). Current therapeutic options for DN include sodium-glucose cotransporter-2 inhibitors, glucagon-like peptide-1 receptor agonists, nonsteroidal mineralocorticoid receptor antagonists, and renin-an giotensin system blockers (Liew et al., 2025). Although these drugs exert certain therapeutic effects, they still have limitations, such as side effects including hyperkalemia and hypotension, as well as the aldosterone escape phenomenon induced by long-term administration, which undermines the therapeutic efficacy. Furthermore, the complex pathological mechanisms of DN itself necessitate the search for safer and more effective therapeutic agents.

Renal fibrosis is an irreversible factor in the progression and deterioration of renal function in DN, marking the final stage of the development of chronic kidney disease towards end-stage kidney disease, and its main pathological feature is the excessive accumulation of extracellular matrix (ECM) (Nogueira et al., 2017; Humphreys, 2018; Zhao et al., 2020). This over - production of ECM components, such as collagen, fibronectin, and laminin, leads to the stiffening and scarring of the renal tissue (Eikmans et al., 2003; Bülow and Boor, 2019). As the fibrotic process advances, the normal architecture and function of the kidneys are gradually compromised, ultimately resulting in a loss of renal function. Thus, understanding the complex interplay between DN and the development of renal fibrosis is essential for developing targeted interventions to slow down or halt the progression of DN.

Inflammation is intricately intertwined with the development of renal fibrosis in DN, a complex and multi - faceted relationship that has been the subject of extensive research in recent years (Rivero et al., 2009; Wada and Makino, 2013). In the context of DN, inflammatory factors and the nucleotide - binding oligomerization domain - like receptor protein 3 (NLRP3) inflammasome emerge as pivotal players, exerting profound influences on both diabetes progression and the onset and advancement of renal fibrosis in DN. The persistent state of hyperglycemia will trigger a chronic inflammatory response in the kidneys. During this process, the released inflammatory mediators serve as initiation signals. These signals are recognized by Toll-like receptors (TLRs) located on the cell surface. Subsequently, this recognition event activates nuclear factor-κB (NF-κB), a key transcription factor involved in the regulation of various immune and inflammatory responses. This series of activation processes promotes the formation of the NLRP3 inflammasome and induces apoptosis through the classical pathway. The rupture and death of cells will trigger extensive inflammatory responses and eventually lead to damage to kidney function (Otto, 2018; Lee et al., 2020). The NLRP3 inflammasome is a multi - protein complex that plays a crucial role in the regulation of inflammation and cell death. Its formation is a highly regulated process that involves the assembly of NLRP3, apoptosis - associated speck - like protein containing a caspase recruitment domain (ASC), and caspase - 1. Once formed, the NLRP3 inflammasome activates caspase - 1, which in turn cleaves pro - interleukin - 1β (pro - IL - 1β) and pro - interleukin - 18 (pro - IL - 18) into their active forms, IL - 1β and IL - 18. These cytokines are potent pro - inflammatory molecules that can further recruit immune cells to the site of inflammation and enhance the inflammatory response (Jo et al., 2016; Kelley et al., 2019). As this vicious cycle of inflammation, apoptosis, and further inflammation continues, the cumulative damage to the renal tissue becomes more and more severe. Studies have shown that the activation of the NLRP3 inflammasome can trigger apoptosis and renal fibrosis, and by inhibiting the activation of the NLRP3 inflammasome, the process of fibrosis in mice can be effectively slowed down (Yang and Zhao, 2023). These findings not only provide valuable insights into the underlying mechanisms of DN but also offer potential therapeutic targets for the prevention and treatment of DN.

*Balanophora involucrata* Hook.f. and Thomson, belongs to the genus Balanophora of the family Balanophoraceae. It is one of the traditional medicines of the Tujia nationality in China, recorded in the Pharmacopoeia of the Tujia Nationality, and was often used to treat kidney diseases and hepatic diseases in ancient times. Balanophora polysaccharide (BPS) is one of the main active ingredients of *Balanophora involucrata*, research has found that BPS can significantly reduce the blood glucose levels of streptozotocin-induced diabetic rats through mechanisms such as antioxidation and improve abnormal lipid metabolism (Chen et al., 2018). Meanwhile, *Balanophora polyandra* (BPP), another specific species of Balanophora, significantly decreases the expression of IL-1β, tumor necrosis factor (TNF-α), BPP also significantly suppresses the activation of NLRP3 inflammasome and the nuclear factor kB (NF-κB). These results suggest that dietary intake of B. polyandra ameliorates colitis (Guo et al., 2020). Moreover, BPP treatment can significantly alleviate interstitial fibrosis through reducing the components (Collagens I, III and IV) of ECM, and reducing the activation of fibroblasts producing these components, as is revealed by inhibiting the hallmarks (fibronectin and α-SMA) of fibroblast activation (Li et al., 2021). However, the impact of BPS on renal injury and renal fibrosis in DN mice is currently unclear.

This study pioneers the exploration of the effects of the natural polysaccharide, BPS, on renal injury and fibrosis in *db/db* mice (a spontaneous diabetic model) with diabetic nephropathy. Remarkably, BPS substantially reduces body weight, along with blood glucose and lipid levels in *db/db* mice. Additionally, it enhances kidney function and effectively mitigates pathological kidney damage. Moreover, BPS plays a crucial role in modulating the ECM - related processes. It reduces the expression of ECM proteins and inhibits ECM deposition, thereby decelerating the progression of DN fibrosis and decreasing podocyte apoptosis. Notably, a correlation exists between the NLRP3 inflammasome and renal fibrosis. BPS effectively decreases the protein expression level of NLRP3, ASC, caspase1, and IL-1β protein in the renal tissue of *db/db* mice, which in turn mitigates the inflammatory response and fibrosis within the kidneys. In conclusion, as a natural product potentially having fewer side effects than synthetic drugs, BPS presents a novel therapeutic option for DN. By decreasing apoptosis, inhibiting inflammation, reducing ECM deposition, and modulating the NLRP3 inflammasome, BPS shows promise in ameliorating renal injury and fibrosis in *db/db* mice. This study holds the promise of promoting the development of BPS - based drugs and their clinical application, offering new hope for patients suffering from this common and challenging complication of diabetes.

## 2 Materials and methods

### 2.1 Preparation and qualitative analysis of *Balanophora involucrata*

*Balanophora involucrata* was collected from Enshi, Hubei Province, and identified as the plant *Balanophora involucrata* (genus Balanophora, family Balanophoraceae) by the Chinese Medicinal Materials Products Quality Supervision and InspectionCenter in Wuling Mountainous Area, Hubei Minzu University.

The phytochemical profiles of *Balanophora involucrata* were carried out with ultrahigh-performance liquid chromatography (UHPLC)-mass spectrometry (MS). The SHIMADZU-LC30 ultra-high-performance liquid chromatography system (UHPLC) was employed, using an ACQUITY UPLC® HSS T3 column (2.1 × 100 mm, 1.8 µm) (Waters, Milford, MA, USA). The column temperature was maintained at 40 °C with a flow rate of 0.3 mL/min. Water containing 0.1% formic acid and acetonitrile solution containing 0.1% formic acid were served as mobile phases A and B, respectively. The chromatographic gradient elution program was as follows: 0–1 min, 0% B; 1–2 min, linear gradient from 0% to 30% B; 2–12 min, linear gradient from 30% to 50% B; 12–21 min, linear gradient from 50% to 100% B; 21–26 min, maintained at 100% B; 26–26.1 min, linear gradient from 100% to 0% B; 26.1–30 min, maintained at 0% B.

TripleTOF 6,600 system (AB Sciex) was used for MS analysis, and the electrospray ionization (ESI) source was used for detection in positive and negative ion modes. The parameters of ESI source were set as follows: the spray voltage was 5.5 kV (+) and 4.5 kV (−), the source temperature was 500 °C, and the range of MS scan was 50–1,500 m/z. The raw mass spectrometry data were processed using MSDIAL software for peak alignment, retention time correction, and peak area extraction. The signal-to-noise ratio of the chromatographic peaks was assessed, and noise background results were filtered out. Compound identification was performed using the TCMSP, HMDB, PubChem, and BPTCM database. Metabolite Structure Identification Parameters: The mass deviation for matching the MS1 was set to mass tolerance <0.01 Da, and for MS2, the mass tolerance was set to <0.02 Da. The total score for identification was required to be > 70.

### 2.2 Preparation and quantitative determination of BPS

**FIGURE 1:**
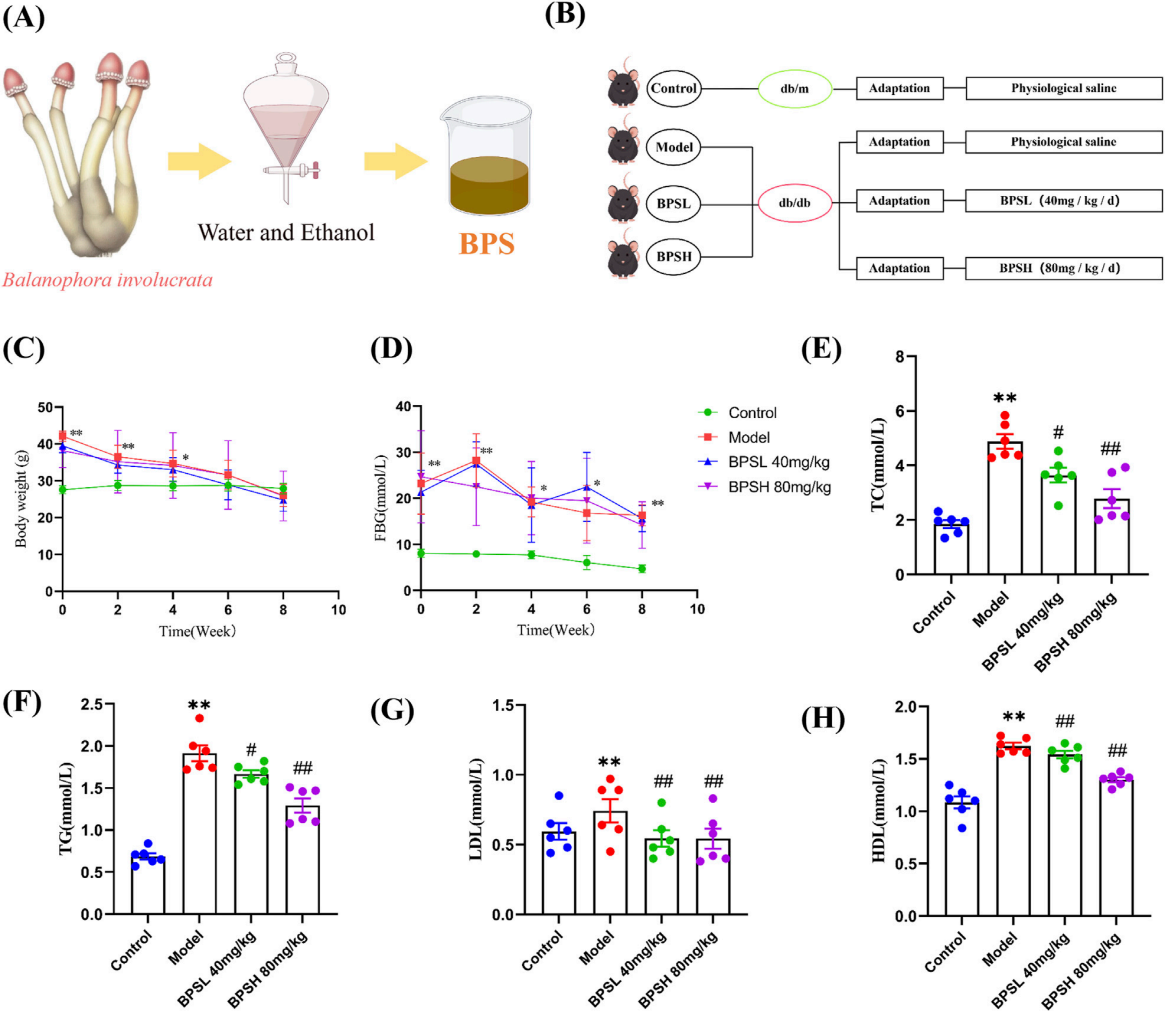
BPS decreases blood glucose and lipid levels of db/db mice. **(A)** Preparation of BPS. **(B)** Experimental design. **(C,D)** Body weight and FBG, n = 8. **(E–H)** Comparison of four blood lipid parameters (TC, TG, LDL, and HDL) in each group of mice, n = 6. Mean ± SD. *P < 0.05, ^**^ *P* < 0.01 vs*.* Control group. ^#^ *P* < 0.05, ^##^ *P* < 0.01 vs*.* Model group.

The drug was dried and pulverized, then soaked in ethanol for 12 h, followed by centrifugation to collect the precipitate. Distilled water was added in a ratio of 20:1, and the mixture was heated for extraction, filtered; this process was repeated twice. The extracts were combined and concentrated. Subsequently, 95% ethanol was added, and the mixture was allowed to stand at 4 °C for 12 h. After centrifugation, the precipitate was collected, freeze-dried, and used for subsequent experiments (Figure 1A).

The anthrone-sulfuric acid method was used for the quantitative determination of BPS. Anhydrous glucose dried to constant weight at 105 °C was used as the reference standard. A reference solution was prepared by dissolving the standard in distilled water. Take 0.1 mL, 0.2 mL, 0.3 mL, 0.4 mL, 0.5 mL, and 0.6 mL of the reference standard solution, respectively, and dilute each to a final volume of 2.0 mL with distilled water. Then, 0.2% anthrone-sulfuric acid reagent was added dropwise to each tube and mixed thoroughly. Absorbance was measured at 582 nm using a UV-Vis spectrophotometer, and a standard curve was constructed by plotting absorbance against glucose concentration.For quantification of BPS, the sample was first dried to constant weight. The same colorimetric procedure applied to the standard curve was then followed for the BPS sample. Finally, the BPS content was determined to be 37.0%.

### 2.3 Animal grouping and drug administration

Twenty-four 8-week-old male specific-pathogen-free (SPF) *db/db* mice and eight db/m mice were purchased from the Jiangsu Laboratory Animal Center (Jiangsu, China). The Animal Certificate Number was NO. SCXK (Jiangsu) 2020–0009. The rearing environment was maintained at a temperature ranging from 24 °C to 26 °C, with a relative humidity of 50%–60%, and a natural alternation of 12 h of light and 12 h of darkness. After 1 week of adaptive feeding, fasting blood glucose of each mouse was measured by taking blood from the tail vein, with a fasting blood glucose level of ≥11.1 mmol/L as the inclusion criterion. The db/m mice were set as the Control group. The *db/db* mice were divided into the model (Model) group, the low-dose balanophora polysaccharide (BPSL 40 mg/kg) group and the high-dose balanophora polysaccharide (BPSH 80 mg/kg) group according to completely randomized design, with eight mice in each group. According to the previous experiments of us (Chen et al., 2018), SD rats were administered with BPS at a dose of 200 mg/kg. Based on the conversion of body surface area between rats and mice, the BPSL group and the BPSH group of *db/db* mice were administered at doses of 40 mg/kg and 80 mg/kg by gavage, respectively. The Control group and the Model group were given the same volume of normal saline, and the administration was carried out once a day for eight consecutive weeks. Figure 1B provides a schematic representation of the experimental design. This experimental protocol was approved by the Medical Ethics Committee of Hubei Minzu University (Ethics Number: 2023077).

### 2.4 Collection of tissue specimens

The body mass and fasting blood glucose of the mice were measured at the 0th, 2nd, fourth, sixth, and eighth weeks of drug administration. Fasting blood glucose was measured by taking blood from the tail vein and records were carefully kept. Before sample collection, the mice were placed in metabolic cages to collect urine for 24 h. Blood was collected from the eyeballs of the mice. After standing still for 2 h, the samples were centrifuged at 14,000 r/min for 20 min at 4 °C to separate and obtain serum. One kidney of each mice was cut in half and then soaked and fixed in 4% paraformaldehyde for 24–36 h before being embedded in paraffin for the preparation of subsequent pathological sections. Another kidney was frozen and stored in a −80 °C freezer for subsequent Western blot detection.

### 2.5 Detection of renal function and four indexes of blood lipids

The contents of urinary creatinine (UCr), 24 - hour urinary protein (24 - hUP), blood urea nitrogen (BUN), triglyceride (TG), total cholesterol (TC), low - density lipoprotein (LDL), and high - density lipoprotein (HDL) were detected using a fully - automatic biochemical analyzer.

### 2.6 Serum inflammatory factors TNF-α and IL-6 detection

The concentrations of serum inflammatory factors TNF-α and IL-6 were evaluated using the ELISA kit (ABclonal Biotechnology Co., Ltd) according to the instructions. Utilize a microplate reader to measure the optical density (OD) values of each well at a wavelength of 450 nm. Subsequently, the levels of TNF-α and IL-6 in each sample were calculated.

### 2.7 Histopathologic evaluation

The isolated renal tissues were fixed in 4% paraformaldehyde for 24–36 h. After dehydration and paraffin embedding, 4 - μm sections were prepared and stained with hematoxylin and eosin (H&E), periodic acid-Schiff (PAS), and Masson trichrome to evaluate the histopathological characteristics. The pathological changes of renal tissues were examined under a microscope (Olympus). The renal fibrosis index was determined by calculating the ratio of the blue area to the total field of vision using Masson trichrome staining.

### 2.8 Glomerular ultrastructure detection by transmission electron microscopy

Tissue blocks with dimensions of 1 mm × 1 mm × 1 mm are taken from the renal cortex of mice and then soaked and fixed in 2.5% glutaraldehyde. Following dehydration and paraffin embedding procedures, ultrathin sections are prepared. These sections are then stained using uranyl acetate and lead citrate staining solutions. Subsequently, the ultrastructure of glomeruli is observed under a transmission electron microscope, and corresponding images are collected.

### 2.9 Cell apoptosis detection by TUNEL assay

TUNEL assay was performed to detect cell apoptosis by using the TUNEL kit (Roche) according to the manufacturer’s instructions. Antigen retrieval was performed with 0.01 M sodium citrate buffer (pH 6.0) at 95 °C–99 °C for 15 min. Endogenous peroxidase activity was blocked with 3% H_2_O_2_ for 10 min at room temperature. After washing with PBS, the TUNEL reaction mixture was added dropwise to cover sections, and incubated at 37 °C in the dark for 60 min. Sections were then incubated with horseradish peroxidase (HRP)-conjugated secondary antibody for 30 min at 37 °C, followed by color development with 3,3′-diaminobenzidine (DAB) and counterstaining with hematoxylin. The number of TUNEL-positive (TUNEL+) cells was counted with a fluorescence microscopy (Olympus) and quantified by using ImageJ software (NIH Image).

### 2.10 Immunohistochemical staining

Immunohistochemical staining Formalin-fixed and paraffin-embedded tissue samples were sliced into 4-µm sections. These sections were then deparaffinized twice with xylene for 10 min each time at room temperature. Subsequently, rehydration was carried out using 100% ethanol twice for 5 min each time, 95% ethanol twice for 2 min each time, and 85% ethanol for 2 min. Following that, the sections were incubated with 3% H2O2 for 10 min to block endogenous peroxidase activity. This was followed by the execution of antigen retrieval in citrate buffer (pH 6.0) at 98 °C for 20 min. Then the sections were incubated with primary antibodies: anti-Collagen Ⅰ (1:400, Immunoway, YM6940), anti-Collagen Ⅳ (1:400, Immunoway, YM6941), or anti-FN (1:100, ZEN-BIOSCIENCE, 250,073), for 1.5 h at room temperature. The slides were then incubated with a biotinylated secondary antibody at 37 °C for 30 min. Subsequently, the reaction products were stained with DAB for 10–20 min and haematoxylin for 2 min. For the blank control, sections were incubated in PBS in the absence of a primary antibody.

### 2.11 Immunofluorescence staining

The sections were prepared in the same manner as described above. For antigen retrieval, the sections were boiled in citrate buffer for 20 min. Subsequently, they were blocked with 5% bovine serum albumin (BSA) for 1 h at room temperature. Then, the sections were immunostained with antibodies against Collagen Ⅳ (1:200, Immunoway, YM6941) in a humid chamber at 4 °C overnight, followed by CY3-labeled goat anti-mouse fuorescent secondary antibody (1:200, SA0000-2, Proteintech) incubation for 1 h. Then, the sections were immunostained with antibodies against NLRP3 (1:500, Proteintech, 68,102-1-1g) in a chamber at 4 °C overnight, followed by 488-labeled goat anti-mouse fuorescent secondary antibody (1:200, Beyotime, A0423) incubation for 1 h. For the co-localization staining of NLRP3 and NF-κB, the procedure is the same as described above, NLRP3 (1:500, Proteintech, 68,102-1-1g) and NF-κB (1:200, Abmart, T55034F). At last, the sections were incubated with DAPI. Fluorescence images were captured by using a fuorescence microscope (Olympus).

### 2.12 Western blot analysis

Total proteins from the renal tissues of mice in each group were extracted using RIPA lysis bufer on ice. Protein concentrations were measured by using a bicinchoninic acid (BCA) protein assay kit (Beyotime). Western blot analysis was performed following a standard protocol. The primary antibodies that were used in this study were as follows: anti-Bax (1:4000, Proteintech, 50,599-2-1g), anti-Bcl-2 (1:2000, Proteintech, 26593-1-AP), anti-caspase 3 (1:5000, Abcam, ab32351), anti-NF-κB (1:1000, Abmart, TA5006), anti-IL-10 (1:5000, Proteintech, 60,269-1-1g), anti-TNF-α(1:1000, Proteintech, 17590-1-AP), anti-TGF-β1 (1:1000, ZEN-BIOSCIENCE, 346,599), anti-α-SMA (1:1000, ZEN-BIOSCIENCE, R23450), anti-TNF-α (1:1000, Proteintech, 17590-1-AP), anti-NLRP3 (1:1000, Proteintech, 68,102-1-1g), anti-ASC (1:1000, Abcam, ab309497), anti-caspase 1 (1:1000, Abcam, ab138483), anti-IL- 1beta (1:1000, Abcam, ab234437) and anti-GAPDH (1:50,000, Proteintech, 60,004-1-1g). The expression of target proteins was normalized to GAPDH obtained from the same sample.

### 2.13 Statistical analysis

Data statistical analysis was performed using IBM SPSS Statistics 25 and GraphPad Prism 9.5.1 software. All data were expressed as mean ± standard deviation (mean ± SD). One-way analysis of variance was used for comparison among multiple groups. *P* value less than 0.05 was considered to indicate a statistically significant difference.

## 3 Results

### 3.1 Chemical composition of *Balanophora involucrata*

Based peak intensity (BPI) chromatograms were shown in Supplementary Figure S1. Preliminary chemical composition analysis revealed that *Balanophora involucrata* contains D-glucose and a variety of carbohydrate derivatives (Table 1), which suggests this medicinal material probably has a rich carbohydrate metabolic basis. To further investigate its macromolecular polysaccharide components, we adopted the water extraction and alcohol precipitation method for extraction, with detailed procedures provided in Section 2.2 “Preparation and Quantitative Determination of BPS”. Finally, the content of BPS was determined to be 37% by the anthrone-sulfuric acid method.

**TABLE 1:** Identification of carbohydrate compounds in Balanophora involucrata.

No.	Compounds	Formula	Delta (PPM)	RT (MIN)	Lonization mode	m/z
1	D-glucose	C_6_H_12_O_6_	1.6196	0.914	[M-H]-	179.05612
2	6-O-galloyl-beta-D-glucose	C_13_H_16_O_10_	1.44986	2.138	[M-H]-	331.06689
3	1-O-Galloyl-beta-D-glucose	C_13_H_16_O_10_	2.02376	3.102	[M-H]-	331.06741
4	1-Caffeoyl-beta-D-glucose	C_15_H_18_O_9_	9.20583	3.519	[M-H]-	341.0881
5	1-O,6-O-Digalloyl-beta-D-glucose	C_20_H_20_O_14_	3.5398	3.519	[M-H]-	483.0777
6	1,2,3-Tri-O-galloyl-beta-D-glucose	C_27_H_24_O_18_	2.97596	3.552	[M-H]-	635.08905
7	1,2,3,6-Tetragalloylglucose	C_34_H_28_O_22_	2.55368	3.643	[M-H]-	787.09991

### 3.2 BPS decreases blood glucose and lipid levels of *db/db* mice

The blood glucose and lipid levels in diabetes are important influencing factors in the onset of DN. So we first evaluated the effects of BPS on body weight and fasting blood glucose of *db/db* mice. Compared with the Control group, the body weight and fasting blood glucose of mice in the Model group were significantly increased (Figures 1C,D, *P* < 0.01). In comparison to the Model group, both the BPSL 40 mg/kg group and the BPSH 80 mg/kg group exhibited a downward trend in body weight and fasting blood glucose (Figures 1C,D). Regarding blood lipid parameters, the levels of TC, TG, LDL, and HDL in the serum of mice in the Model group were significantly elevated compared to those in the Control group (Figures 1E–H, *P* < 0.01). While compared with the Model group, the contents of TC, TG, LDL, and HDL in the serum of mice in the BPSL 40 mg/kg group and the BPSH 80 mg/kg group were significantly decreased (Figures 1E–H, *P* < 0.05). These findings indicate that BPS effectively decreases blood glucose and lipid levels of *db/db* mice.

### 3.3 BPS improves the renal function while alleviates pathological renal injury in *db/db* mice

**FIGURE 2:**
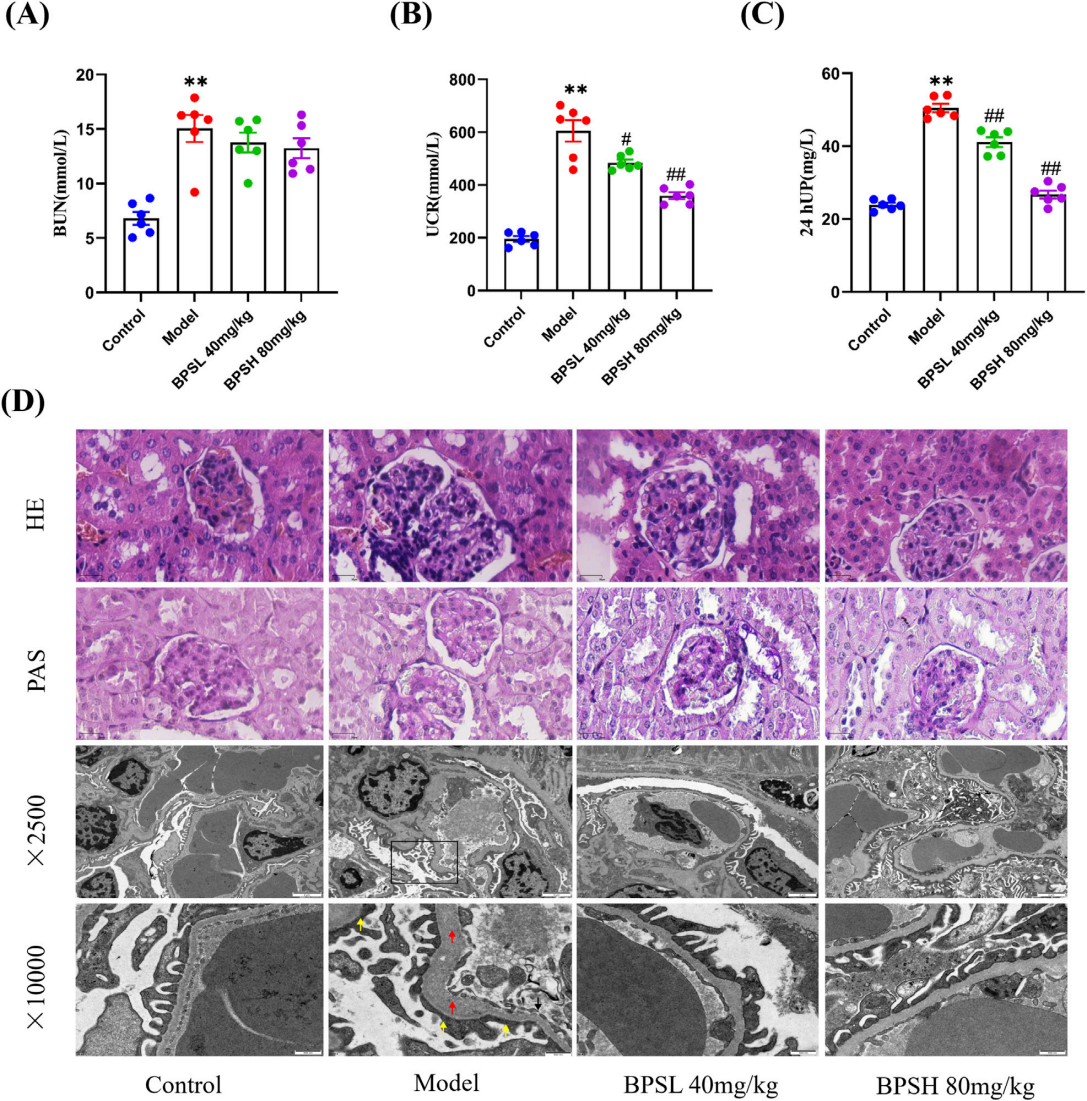
BPS improves the renal function while alleviates pathological renal injury in *db/db* mice. **(A-C)** Comparison of three renal function indexes (BUN, UCr, and 24 hUP) in each group of mice, n = 6. **(D)** Pathological changes of renal tissue in each group of mice by HE staining. scale bar, 50μm, n = 5. Glycogen accumulation of renal tissue in each group of mice by PAS staining. scale bar, 50μm, n = 5. Ultrastructural observation of mouse glomeruli in each group of mice, n = 3. black box indicates empty capillary endothelium, red arrow indicates local basement membrane thickening, loose and swollen, and yellow arrow indicates large fusion of foot processes, flattened adhesion and reduced holes. scale bar, 2 μm (above); scale bar, 500 nm (below).

We examined the effects of BPS on renal function indexes of *db/db* Mice. When compared to the Control group, the levels of BUN, UCr, and 24 hUP in the mice of the Model group were significantly elevated (Figures 2A–C, *P* < 0.01). In contrast, when compared with the Model group, the levels of UCr and 24 hUP in the mice of the BPSL 40 mg/kg group with BPSH 80 mg/kg group were significantly reduced (Figures 2A–C, *P* < 0.01 and *P* < 0.05). Additionally, the level of BUN in these two groups showed a downward trend; however, the difference was not statistically significant (Figures 2A–C, *P* > 0.05).

The HE staining results presented in Figure 2D indicated that, when compared to the Control group, the glomeruli volume of mice in the Model group was enlarged, the basement membrane was thickened, inflammatory cell infiltration could be seen around, and the structure of renal tubules was disordered and accompanied by dilation. In contrast, when compared with the Model group, the mice in the BPSL 40 mg/kg group and the BPSH 80 mg/kg group demonstrated varying degrees of improvement in terms of glomerular volume, basement membrane thickness, and the extent of inflammatory cell infiltration (Figure 2D).

The impact of BPS on glycogen accumulation within the renal tissues of *db/db* mice was also assessed via PAS staining. As depicted in Figure 2D, when contrasted with the Control group, the Model group exhibited a thickened basement membrane of the glomeruli, an accumulation of extracellular matrix, and a substantial deposition of purplish - red glycogen within the renal capsule. Conversely, in comparison to the Model group, the BPSL 40 mg/kg group and the BPSH 80 mg/kg group showed a decrease in the glycogen deposition in the renal capsule of the mice (Figure 2D).

The effect of BPS on the ultrastructure of glomeruli of *db/db* mice was also detected by transmission electron microscopy. The findings revealed that, as opposed to the Control group, the glomerular filtration membrane of mice in the Model group exhibited an empty capillary endothelium (Figure 2D). Simultaneously, the local basement membrane underwent thickening, accompanied by a state of looseness and swelling. Moreover, a substantial number of foot processes underwent fusion, adhering to one another and flattening, thereby resulting in a reduction in the number of slit pores (Figure 2D). However, when compared to the Model group, the ultrastructure of glomeruli in mice of the BPSL 40 mg/kg group and the BPSH 80 mg/kg group demonstrated varying degrees of improvement (Figure 2D).

Collectively, BPS effectively improves the renal function of *db/db* mice.And the results of HE staining, PAS staining, and transmission electron microscopy indicate that BPS alleviates pathological renal injury in *db/db* mice.

### 3.4 BPS reduces the expression of ECM proteins, inhibits ECM deposition, and delays the progression of fibrosis in *db/db* mice

**FIGURE 3:**
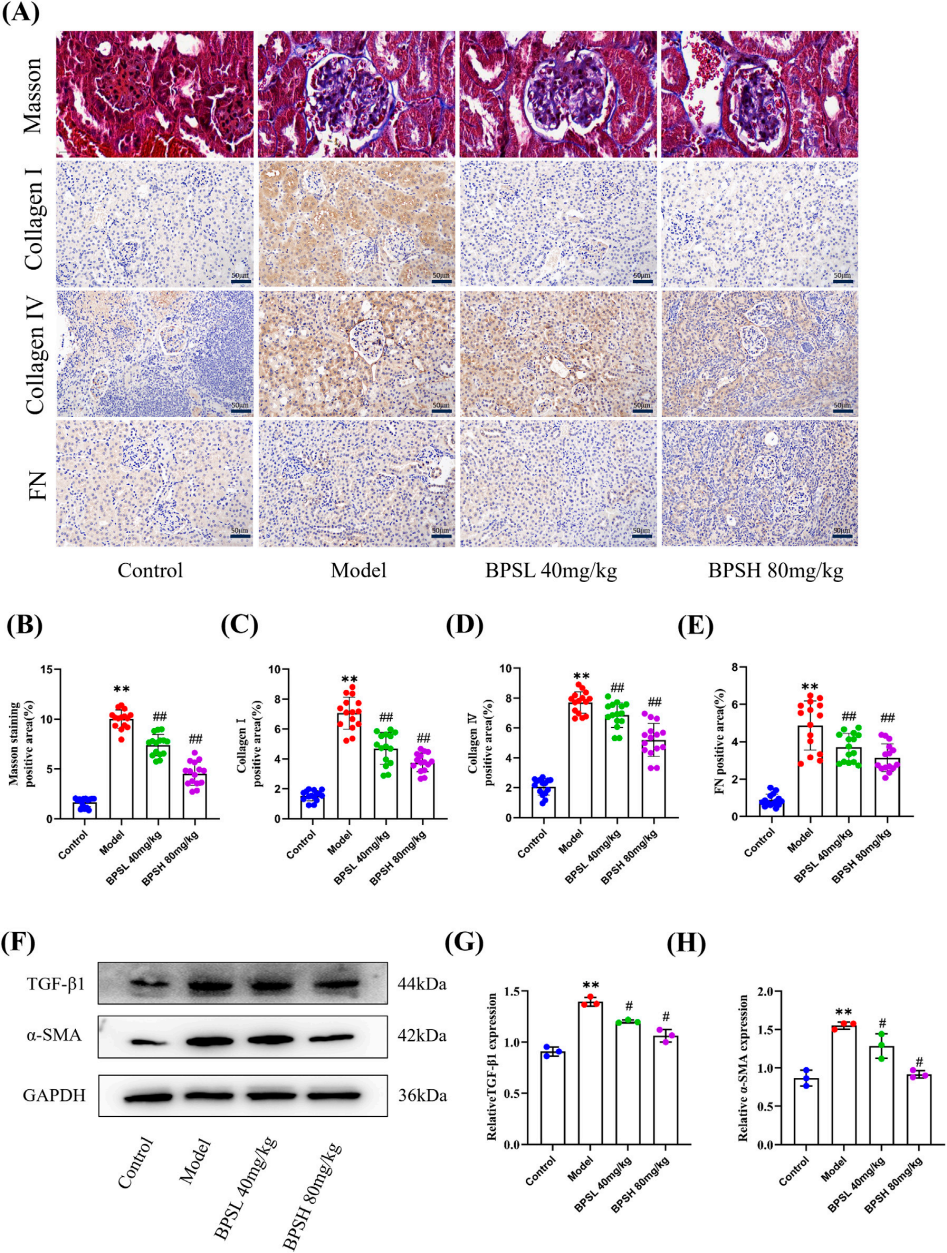
BPS reduces the expression of ECM proteins, inhibits ECM deposition, and delays the progression of fibrosis in *db/db* mice. **(A)** Representative images of masson’s trichrome stain of renal tissue and protein expression of Collagen Ⅰ, Collagen Ⅳ, and FN by immunohistochemical staining in each group of mice. scale bar, 50 μm. **(B–E)** The statistical analyses of the average percentage of Masson’s trichrome stained positive cells and the protein expression levels (positive areas) of Collagen Ⅰ, Collagen Ⅳ, and FN. **(F)** Expression of TGF-β1 and α-SMA proteins in the renal tissue of mice in each group as determined by Western blot. Antibody against GAPDH is used as an internal control. **(G,H)** The quantitative analysis for the relative protein expression levels of TGF-β1 and α-SMA based on Western blot results. For pathological staining, five different areas from the kidney images of three mice were randomly selected for statistics. Mean ± SD. ^**^ *P* < 0.01 vs*.* Control group. ^##^ *P* < 0.01 vs*.* Model group. ^#^ *P* < 0.05 vs*.* Model group.

The results of Masson staining showed that, compared with the Control group, a large amount of collagen could be seen in the renal interstitial tissues of mice in the Model group, and the interstitial fibrous tissues showed a proliferation phenomenon in bundles and networks (Figure 3A). However, upon the administration of BPS, the progression of fibrosis was decelerated as evidenced by a reduction in collagen fibers observed in the mice of BPSL 40 mg/kg group and the BPSH 80 mg/kg group (Figure 3B).

The pivotal mechanism underlying renal fibrosis is the transdifferentiation process of renal tubular epithelial cells. During this intricate process, the ECM undergoes abnormal synthesis. Consistently, immunohistochemical findings revealed that, when contrasted with the Control group, the renal tissues of *db/db* mice exhibited augmented positive expressions of Collagen Ⅰ, Collagen Ⅳ, and FN, which served as the key fibrosis indices closely associated with ECM synthesis (Figure 3A). Conversely, following BPS intervention, the expression levels of these fibrosis-associated proteins in both the BPSL 40 mg/kg group and the BPSH 80 mg/kg group declined (Figures 3C–E). Correspondingly, the staining appeared lighter. Meanwhile, the Western blot results showed that the expression levels of fibrosis-related proteins TGF-β1 and α-SMA were increased in the model group, and decreased after BPS treatment when compared with the normal group (Figures 3F–H, *P* < 0.05).

This compellingly demonstrates that BPS is capable of reducing the expression of ECM proteins, suppressing ECM deposition, and thereby retarding the progression of fibrosis in DN.

### 3.5 BPS reduces cell apoptosis in *db/db* mice

**FIGURE 4:**
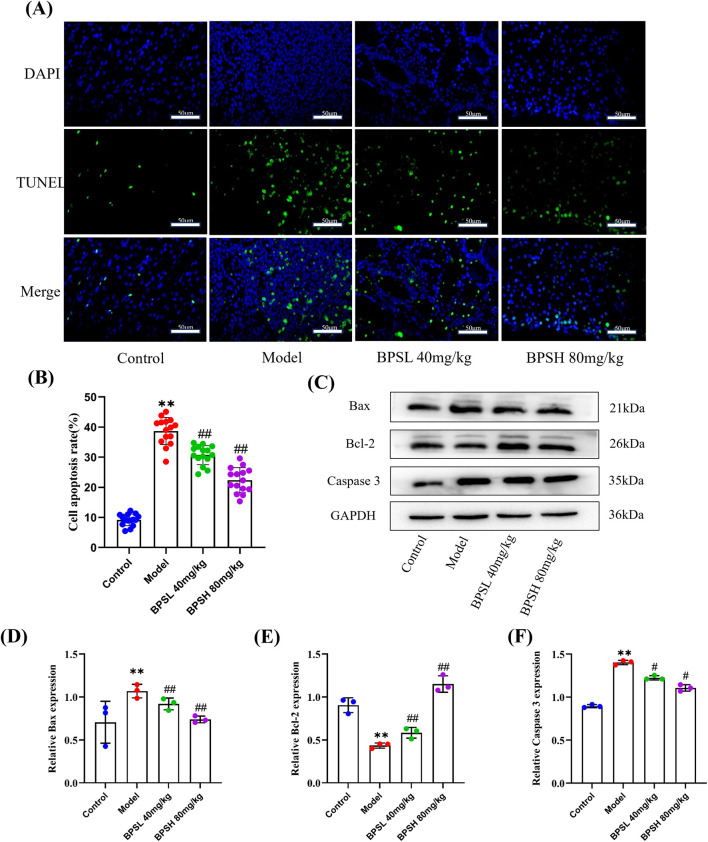
BPS reduces cell apoptosis in *db/db* mice. **(A)** Cell apoptosis in the kidney in mice in each group. Representative images of TUNEL staining of renal tissue in each group of mice. scale bar, 50 μm. **(B)** The statistical analyses of the average percentage of apoptosis in the kidney. **(C–F)** Expression of Bax, Bcl-2 and Caspase 3 proteins in the renal tissue of mice in each group as determined by Western blot. Antibody against GAPDH is used as an internal control. For fluorescence tests, five different areas from the kidney images of three mice were randomly selected for statistics; For WB experiments, n = 3. Mean ± SD. ^**^ *P* < 0.01 vs*.* Control group. ^#^ *P* < 0.05, ^##^ *P* < 0.01 vs*.* Model group.

We investigated the effect of BPS on cell apoptosis in *db/db* mice. The results of TUNEL staining demonstrated that, compared with the Control group, the green fluorescence intensity in the glomerular area of the Model group was significantly elevated, and the number of TUNEL+ cells increased substantially (Figures 4A,B, *P* < 0.01). This indicated a marked increase in cell apoptosis. Conversely, in the BPSL 40 mg/kg and BPSH 80 mg/kg groups, the number of TUNEL+ cells in the glomerular area of mice decreased, and the fluorescence intensity was significantly attenuated (Figure 4A, *P* < 0.01). These findings suggest that BPS alleviates cell apoptosis. In addition, the results of Western blot further demonstrated that the expression levels of apoptosis-related proteins in the Model group, including Bax and Caspase 3, were significantly higher than that in the Control group (Figures 4C–F, *P* < 0.01). Conversely, when compared with the Model group, the protein expression leves of Bax and Caspase 3 in the BPSL 40 mg/kg group and the BPSH 80 mg/kg group were markedly decreased (Figures 4C–F, *P* < 0.01). At the same time, the protein expression of Bcl-2 was increased significantly in the BPSL 40 mg/kg group and the BPSH 80 mg/kg group when compared with the Model group (Figures 4C–F, *P* < 0.01).

### 3.6 BPS alleviates inflammation and regulates the fluorescent colocalization of NLRP3 and collagen Ⅳ in *db/db* mice

**FIGURE 5:**
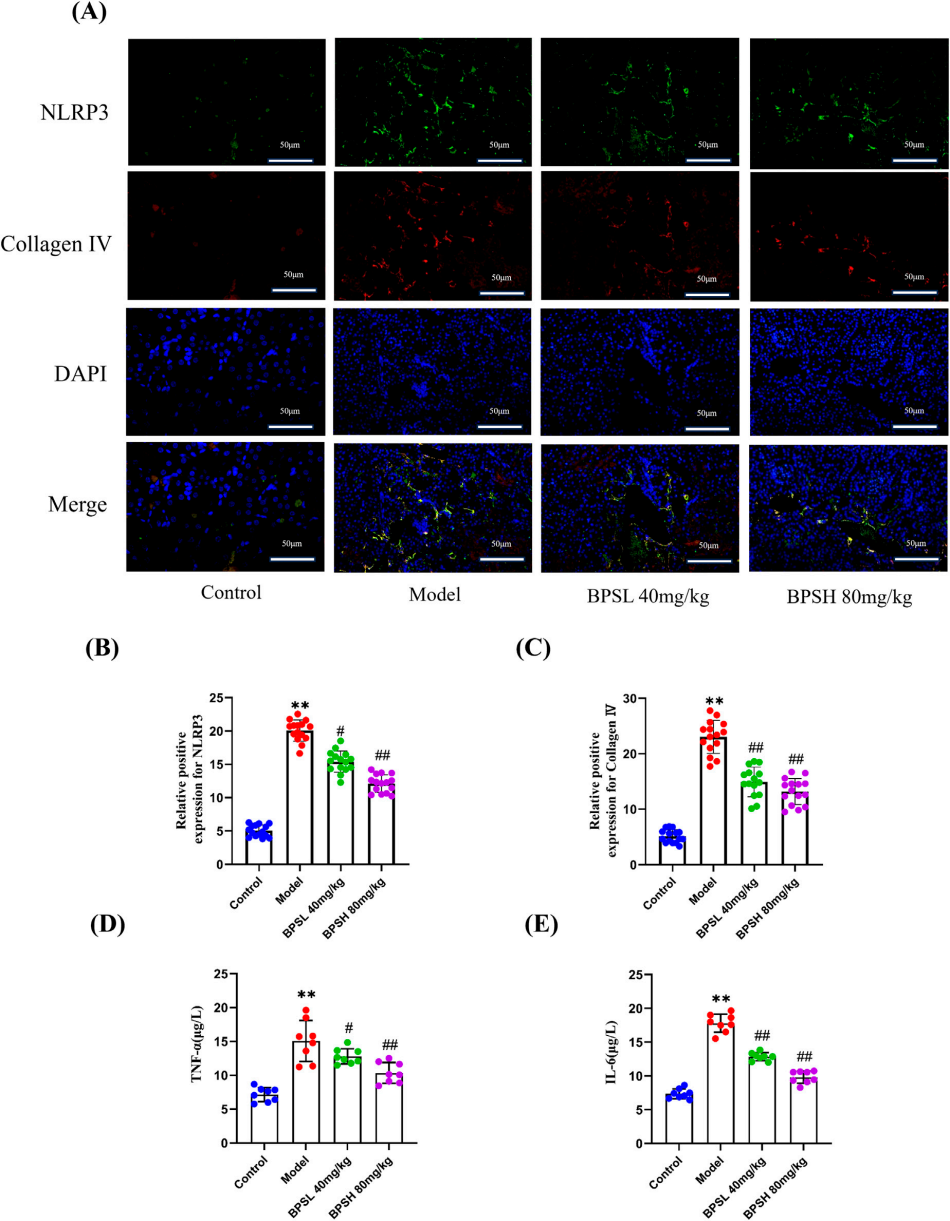
BPS alleviates inflammation and regulates the fluorescent colocalization of NLRP3 and Collagen four in db/db mice. **(A)** Colocalized expression of NLRP3 and Collagen Ⅳ in the renal tissue of mice in each group as determined by immunofluorescence. **(B)** Statistical analysis of the positive expression of NLRP3. **(C)** Statistical analysis of the positive expression of Collagen Ⅳ **(D)** Comparison of serum levels of TNF-α in each group of mice, n = 8. **(E)** Comparison of serum levels of IL-6 in each group of mice, n = 8. For fluorescence tests, five different areas from the kidney images of three mice were randomly selected for statistics. Mean ± SD. ^**^ *P* < 0.01 vs*.* Control group. ^#^ *P* < 0.05, ^##^ *P* < 0.01 vs*.* Model group.

Immunofluorescence results showed that there was co-localization of NLRP3 and Collagen Ⅳ in renal tissues of db/db mice in the model group (Figures 5A–C). However, after BPS treatment, compared with the Model group, the co-localization of NLRP3 and Collagen Ⅳ in renal tissues of db/db mice in the BPSL 40 mg/kg group and BPSH 80 mg/kg group showed a decreasing trend, and the expression of NLRP3 and Collagen Ⅳ was decreased, suggesting a possible correlation between the NLRP3 inflammasome and Collagen Ⅳ (Figures 5A–C).

In addition, Inflammation is closely related to the development of renal fibrosis in DN. Our findings revealed that the serum levels of TNF-α and IL-6 in the Model group of mice were significantly elevated compared to those in the Control group (Figures 5D,E, *P* < 0.01). In contrast, in both the BPSL 40 mg/kg group and the BPSH 80 mg/kg group of *db/db* mice, BPS effectively decreased the serum levels of TNF - α and IL-6 (Figures 5D,E, *P* < 0.05 and *P* < 0.01). This indicates that BPS has the potential to mitigate the inflammatory response in the kidneys of *db/db* mice. Consistently, Western blot results demonstrated that the protein expression level of TNF-α in the Model group was significantly higher than that in the Control group (Figures 7A,E, *P* < 0.01). Conversely, when compared with the Model group, the protein expression levels of TNF-α in the BPSL 40 mg/kg group and the BPSH 80 mg/kg group were markedly decreased (Figures 7A,E, *P* < 0.01 and *P* < 0.05).

### 3.7 BPS regulates the fluorescent colocalization of NLRP3 and NF-κB in *db/db* mice

**FIGURE 6:**
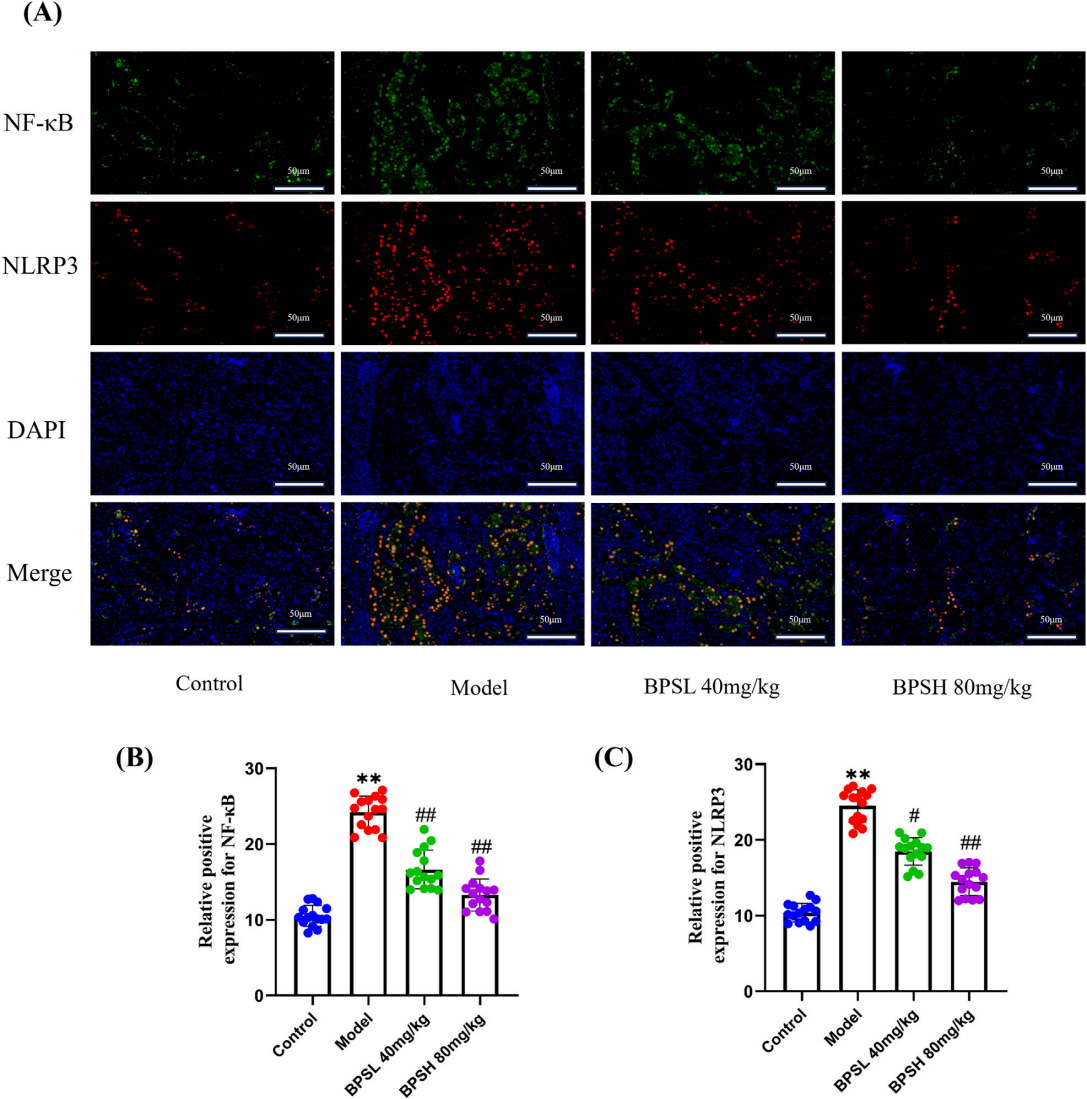
BPS regulates the fluorescent colocalization of NLRP3 and NF-κB in db/db mice. **(A)** Colocalized expression of NLRP3 and NF-κB in the renal tissue of mice in each group as determined by immunofluorescence. **(B)** Statistical analysis of the positive expression of NF-κB. **(C)** Statistical analysis of the positive expression of NLRP3. For fluorescence tests, five different areas from the kidney images of three mice were randomly selected for statistics. Mean ± SD. ^**^ *P* < 0.01 vs*.* Control group. ^#^ *P* < 0.05, ^##^ *P* < 0.01 vs*.* Model group.

**FIGURE 7:**
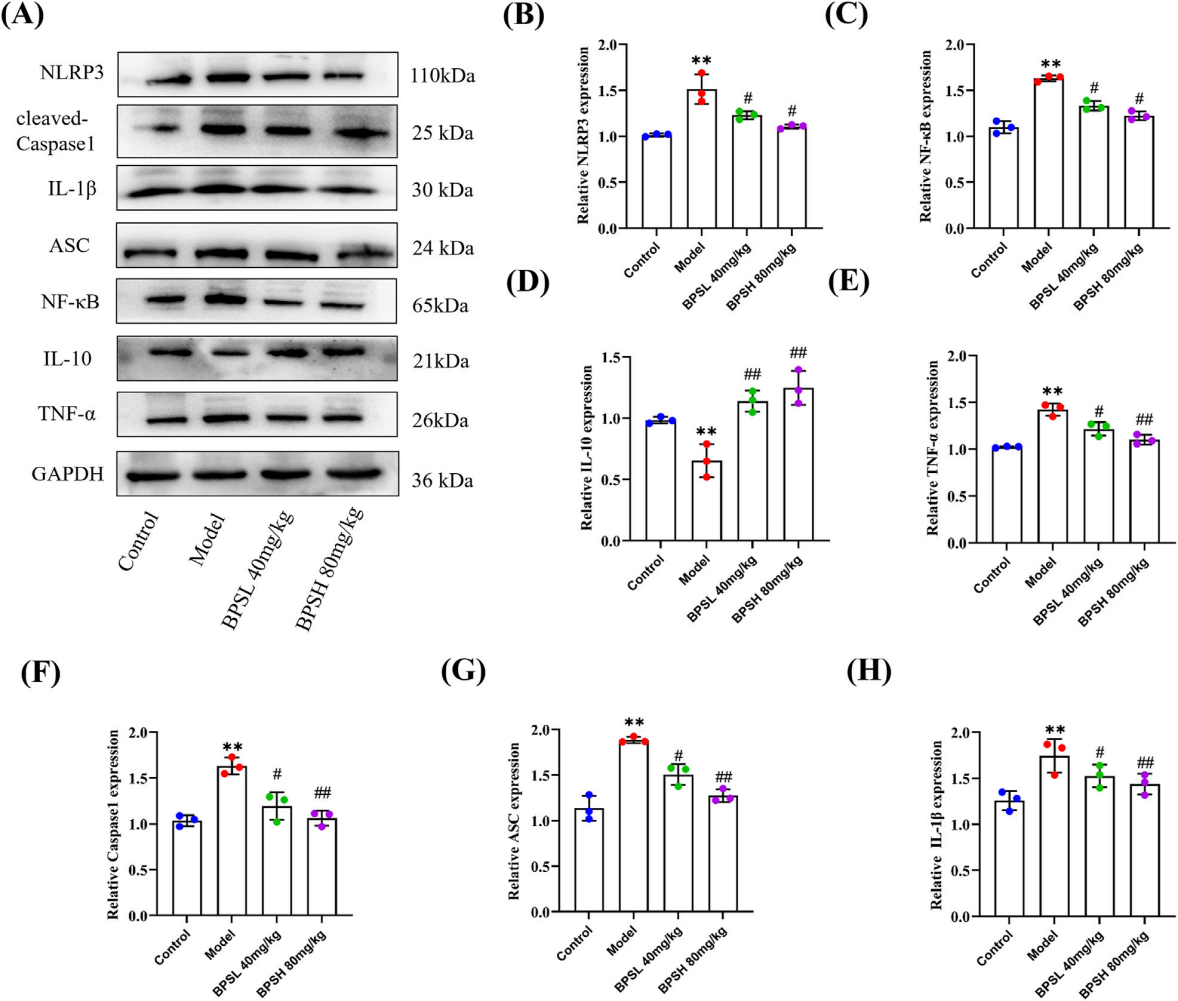
BPS may alleviate inflammation and renal fibrosis in *db/db* mice by regulating NLRP3 inflammasome. **(A)** Expression of NLRP3, NF-κB, IL-10, TNF-α, ASC, IL-1β, and Caspase1 proteins in the renal tissue of mice in each group as determined by Western blot. Antibody against GAPDH is used as an internal control. **(B–H)** The quantitative analysis for the relative protein expression levels of NLRP3, NF-κB, IL-10, TNF-α, ASC, IL-1β, and Caspase1 based on Western blot results. n = 3, Mean ± SD. ^**^ *P* < 0.01 vs*.* Control group. ^#^ *P* < 0.05, ^##^ *P* < 0.01 vs*.* Model group.

Immunofluorescence results showed that there was co-localization of NLRP3 and NF-κB in renal tissues of db/db mice in the model group (Figures 6A–C). However, after BPS treatment, compared with the Model group, the co-localization of NLRP3 and NF-κB in renal tissues of db/db mice in the BPSL 40 mg/kg group and BPSH 80 mg/kg group showed a decreasing trend, and the expression of NLRP3 and NF-κB was decreased, suggesting a possible correlation between the NLRP3 inflammasome and NF-κB (Figures 6A–C). According to Figure 7, BPS may inhibit the NLRP3 inflammasome by suppressing the NF-κB pathway.

### 3.8 BPS may alleviate inflammation and renal fibrosis in *db/db* mice by regulating NLRP3 inflammasome

The NLRP3 inflammasome is a potent inflammatory mediator. During the process of fibrosis, the inflammasome plays a crucial role. Previous studies have shown that NLRP3 regulates renal fibrosis and inflammation by modulating TGF-β1 (Fu et al., 2017). In this study, Western blot results indicated that NLRP3, ASC, IL-1β, and Caspase1 were significantly upregulated in the renal tissues of *db/db* mice in the Model group (Figures 7A–H, *P* < 0.01). Consistent with the expression trend, the expressions of NF-κB and the inflammatory factor TNF-α were also upregulated, but IL-10 was significantly reduced (Figures 7A–H, *P* < 0.01). Moreover, TGF-β1 and α-SMA, the important component of the ECM, were also upregulated accordingly (Figures 3F–H, *P* < 0.01). However, after treatment with BPS, the protein expression levels of NLRP3, NF-κB, TNF-α, ASC, IL-1β, and Caspase1 in the renal tissues of *db/db* mice in the BPSL 40 mg/kg group and BPSH 80 mg/kg group were all downregulated, but IL-10 was significantly upregulated (Figures 7A–H, *P* < 0.01 and *P* < 0.05). Meanwhile, the expressions of fibrotic proteins TGF-β1 and α-SMA were decreased (Figures 3F–H, *P* < 0.05).

In conjunction with Figures 5, 7, these results demonstrate that BPS can regulate the protein expression level of the NLRP3 inflammasome. There is a certain relationship between the NLRP3 inflammasome and renal fibrosis. BPS may alleviate the inflammatory response and the process of renal fibrosis in *db/db* mice by regulating NLRP3 inflammasome.

## 4 Discussion

DN is one of the most common microvascular complications of diabetes mellitus, mainly manifested as changes in kidney structure and function damage caused by diabetes. The course of diabetes, blood glucose level, blood pressure level, and blood lipids are the influencing factors for the onset of DN (Li et al., 2020). The pathogenesis of DN is complex, and its exact mechanism has not yet been clearly defined. Current research results suggest that multiple factors such as metabolic disorders, hemodynamic changes, inflammatory response mechanisms, cytokines, oxidative stress, genetic factors, the kinin system, and autophagy are involved in the pathogenesis of DN (Kanwar et al., 2011; Wada and Makino, 2013). Recent studies have confirmed that NLRP3 plays a crucial role in the pathological progression of DN (Guo et al., 2025). Specifically, NLRP3 can be activated through multiple pathways, mediates pyroptosis and inflammation, and thereby promotes the progression of DN (Wan et al., 2022). These activating pathways include mitochondria, oxidative stress, and neutrophil extracellular traps (NETs), among others (Han et al., 2018; Gupta et al., 2022; Qi et al., 2023). Traditional Chinese medicine in the treatment of DN shows the synergistic effects of multiple targets and multiple pathways, with few side effects and high safety. For instance, Calycosin can ameliorate DN via multiple pathways, such as RAGE, NLRP3 inflammasome, and Nrf2, among others (Dalal et al., 2025). Astragalus mongholicus polysaccharides, Sanziguben polysaccharides, and other polysaccharides can target the NLRP3 inflammasome to ameliorate DN, which demonstrates the potential of polysaccharides in treating DN by inhibiting the NLRP3 inflammasome (Wang et al., 2023; Xu et al., 2025).

The db/db mice are a spontaneous diabetic model, characterized by obesity and persistent hyperglycemia. After 20 weeks of age, they may develop DN (Suriano et al., 2021). In this study, we found that BPS can reduce the body weight and fasting blood glucose of db/db mice, decrease the contents of renal function indexes, such as UCr, 24 hUP, and BUN, and improve the levels of TC, TG, LDL, and HDL in the serum, indicating that BPS can reduce the blood glucose and blood lipid levels of db/db mice and improve renal function. The main pathological features of DN are thickening of the glomerular basement membrane, expansion of the glomerular mesangium, glomerulosclerosis, and tubulointerstitial fibrosis (Wang et al., 2020; Jing et al., 2021). Through pathological examinations, it was found that in the renal tissues of mice in the Model group, obvious enlargement of the glomerular volume, thickening of the basement membrane, increased glycogen deposition, a large number of collagen fibers, and fusion of podocytes could be seen. After BPS intervention, the pathological kidney damage of db/db mice could be alleviated, and the fibrotic progress of DN could be delayed.

In diabetic nephropathy, factors such as high blood glucose disrupt the balance between Bax and Bcl-2, leading to a relative increase in Bax and a relative decrease in Bcl-2. This imbalance activates Caspase 3, ultimately resulting in cell apoptosis (Lee et al., 2009; Liu et al., 2021). After cell apoptosis, their normal physiological functions are lost, the glomerular filtration barrier is damaged, and a large amount of protein is lost in the urine. This further trigger pathophysiological changes in the kidneys and promotes the occurrence and development of diabetic nephropathy. We found that BPS can reduce the levels of Bax and caspase 3, leading to a relative increase in Bcl-2 in the db/db mice, indicating that BPS can alleviate the cell apoptosis in the kidneys of db/db mice. Inflammatory factors are closely related to the pathogenesis of DN. During DN, macrophages and lymphocytes will produce inflammatory factors such as IL-6 and TNF-α, thus forming an inflammatory state in the kidneys (Chen et al., 2022a; Yang and Zhang, 2024). We found that BPS can reduce the levels of inflammatory factors TNF-α and IL-6 in the serum of db/db mice, indicating that BPS can alleviate the inflammatory response in the kidneys of db/db mice.

Renal fibrosis refers to the situation where the kidneys are stimulated by multiple pathogenic factors, resulting in damage to resident cells. In the later stage, a large amount of collagen deposition and accumulation occur, causing the renal parenchyma to gradually harden, form scars, and eventually lead to the complete loss of organ function of the kidneys. This is the main pathological factor of DN (Zanchi et al., 2017). The core mechanism of renal fibrosis is the trans differentiation process of renal tubular epithelial cells. This process is usually initiated under the action of pathological factors such as glycosuria, proteinuria, oxidative stress, and inflammation. Some renal tubular epithelial cells, in order to resist environmental damage and avoid potential apoptosis, transform into myofibroblasts through phenotypic transformation and migrate into the interstitial, abnormally synthesizing ECM (Suriano et al., 2021). ECM is mainly composed of α-SMA, Collagen Ⅰ, Collagen Ⅳ, and FN (Zheng et al., 2019). And TGF-β1 plays an important role in the process of fibrosis, guiding fibrosis through multiple cell types and multiple interacting signal pathways (Abbate et al., 2002; Kim et al., 2018). We found that after BPS intervention, the protein expression levels of fibrosis indexes such as Collagen Ⅰ, Collagen Ⅳ, FN, α-SMA, and TGF-β1 in the renal tissues of db/db mice decreased, indicating that BPS can reduce the protein expression of ECM, inhibit ECM deposition, and thus delay the fibrotic progress of DN.

The NLRP3 inflammasome is an effective inflammatory mediator. Recent studies have found that it shows significant efficacy in activating the sterile inflammatory response in DN (Qiu and Tang, 2016; Fu et al., 2017). It is reported that knocking out the NLRP3 gene can effectively inhibit the infiltration of macrophages in the kidneys of DM rats, reduce the protein expression of TGF-β1 and CTGF, improve glomerular lesions in mice, and reduce the protein expression of FN, Collagen Ⅰ, and Collagen Ⅳ(Wu et al., 2018). As an important inflammatory regulatory complex, the NLRP3 inflammasome plays a central role in the inflammatory response to DN. Hyperglycemia induces the assembly and activation of the NLRP3 inflammasome, prompting the release of numerous inflammatory factors, such as IL-1β and IL-18 (Chen et al., 2025). These inflammatory factors will further aggravate the inflammatory damage of the kidney tissue, attracting inflammatory cells to infiltrate into the kidney, forming a vicious cycle, leading to the continuous aggravation of the kidney damage. Meanwhile, the NF-κB signaling pathway also plays a key role in the inflammatory process of DN (Li et al., 2022; Yosri et al., 2022). Hyperglycemic stimulation can activate the NF-κB signaling pathway to enter the nucleus and regulate the transcription and expression of a series of inflammation-related genes, and then promote the synthesis and release of inflammatory factors and fibrosis-related factors such as TNF-α, IL-6, TGF-β one and α-SMA. TNF-α and IL-6 are cytokines with potent pro-inflammatory effects, which are able to enhance vascular permeability, promote the adhesion and migration of inflammatory cells, and aggravate the inflammatory response of kidney tissues (Xiang et al., 2020; Qiu et al., 2023) In addition, the NF-κB pathway exerts a regulatory effect on the NLRP3 inflammasome. Results from immunofluorescence double staining and Western blot analysis showed that the regulatory effect of BPS on inflammation and fibrosis may be exerted by inhibiting the NLRP3 inflammasome through the NF-κB pathway. TGF-β1, as a key molecule linking inflammation and fibrosis, exerts dual “pro-inflammatory” and “pro-fibrotic” effects. On one hand, it can activate the NF-κB signaling pathway, thereby further amplifying inflammatory responses. On the other hand, it directly stimulates the activation of renal interstitial fibroblasts and promotes the synthesis and deposition of ECM. This dual role makes the persistent activation of inflammation a critical inducer for the initiation of renal fibrosis. The elevated expression of α-SMA, a landmark protein of myofibroblasts, implies increased activation and proliferation of myofibroblasts in the kidney, which leads to excessive production and deposition of ECM and accelerates the progression of renal fibrosis (Chen et al., 2022b; Venugopal et al., 2022). In this study, we observed significantly increased levels of inflammatory factors TNF-α and IL-6 in serum of db/db mice, we also found the activation of NLRP3 inflammasome and NF-κB signaling, which resulted in significantly increased expression levels of inflammatory factors such as TNF-α, TGF-β one and α-SMA. The expression levels of these indicators decreased significantly after BPS intervention, which fully demonstrated that BPS can effectively inhibit inflammation and reduce the damage of inflammation to the kidney, and thus delay the progression of DN. Moreover, IL-10, as an anti-inflammatory cytokine, was increased in db/db mice after BPS intervention, indicating that BPS can regulate the immune balance, enhance the body’s anti-inflammatory ability, help fight the inflammatory response and protect kidney function. Furthermore, the number of co-localization positive cells of the NLRP3 inflammasome and the fibrosis index Collagen Ⅳ reduce, indicating that BPS can regulate the protein expression level of the NLRP3 inflammasome and there is a certain relationship between the NLRP3 inflammasome and renal fibrosis.

Compared with NLRP3 inhibitors (e.g., MCC950, OLT1177) and anti-inflammatory drugs for DN, BPS exhibits distinct advantages, including multi-target and synergistic properties, as well as lower cost and relatively fewer side effects. However, BPS has not yet undergone large-sample animal experiments or clinical controlled trials, thus cannot match drugs such as NLRP3 inhibitors and classic anti-inflammatory agents—which possess clear target specificity and wide clinical application. Additionally, as a bioactive component derived from traditional Chinese medicine, the potential off-target effects and safety/toxicity profiles of BPS necessitate further evaluation. From the perspective of off-target effects, although BPS primarily targets inflammatory pathways (e.g., the NLRP3 inflammasome) and fibrotic mediators (e.g., TGF-β1), it may inadvertently interact with non-target signaling cascades—for instance, it could non-specifically modulate other innate immune pathways. Therefore, further studies using metabolomics, proteomics, and other approaches are required to clarify its exact targets. Furthermore, *Balanophora involucrata* itself is non-toxic; nevertheless, additional research is still needed to assess the safety and toxicity of its extract, BPS.

## 5 Conclusion

**FIGURE 8:**
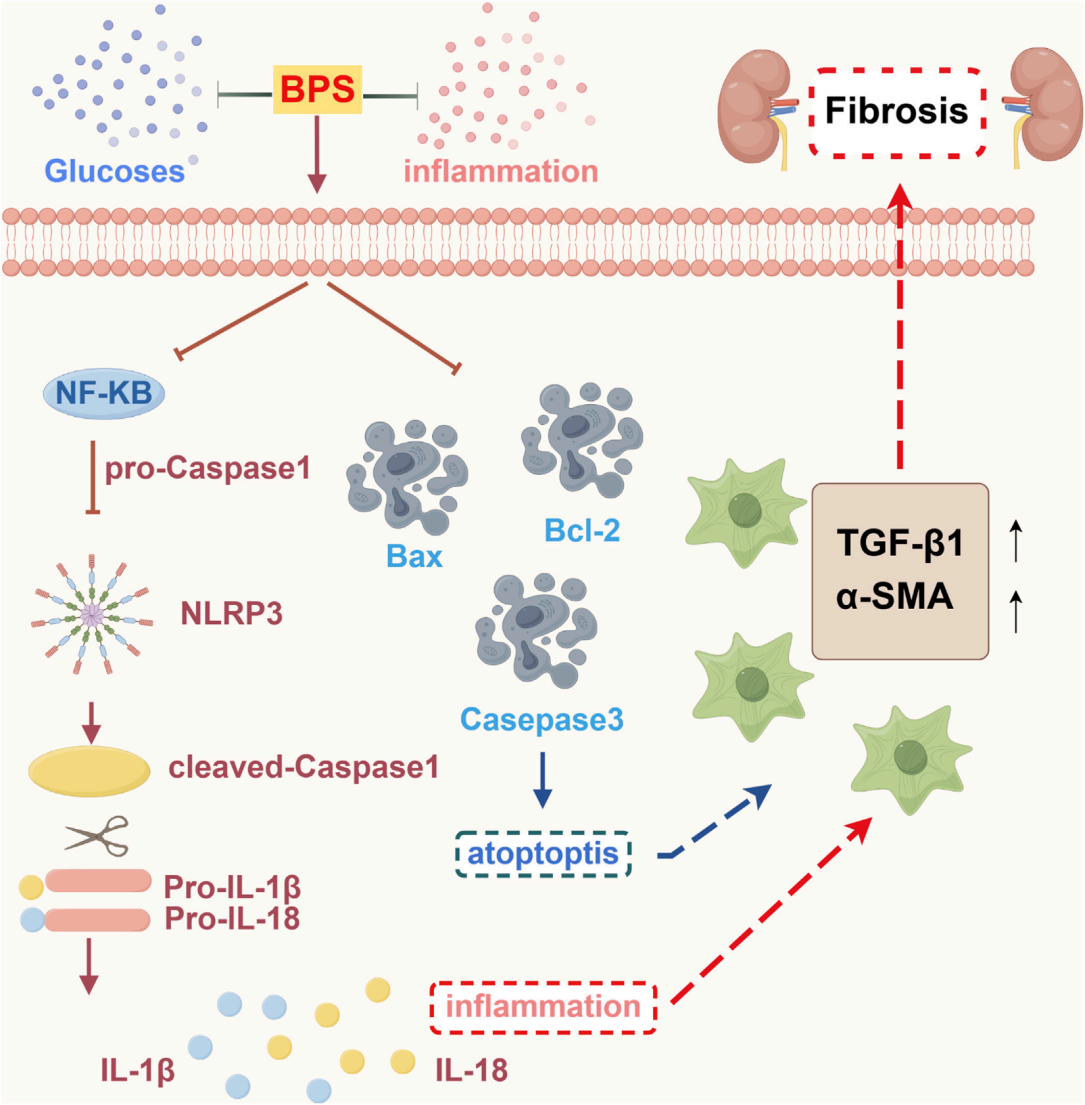
Schematic diagram of the potential mechanisms of BPS against DN.

In summary, this study reveals that BPS can improve kidney damage and renal fibrosis in *db/db* mice with DN, and its mechanism may be related to reduced apoptosis, inhibiting inflammation, reducing ECM deposition, and regulating the NLRP3 inflammasome (Figure 8). Meanwhile, this study provides a modern biological basis for the clinical application of BPS in the treatment of renal fibrosis in DN and is expected to promote the drug development and clinical application of BPS.

## Data Availability

The raw data supporting the conclusions of this article will be made available by the authors, without undue reservation.

## References

[B1] Abbate M. Zoja C. Rottoli D. Corna D. Tomasoni S. Remuzzi G. (2002). Proximal tubular cells promote fibrogenesis by TGF-beta1-mediated induction of peritubular myofibroblasts. Kidney Int. 61, 2066–2077. 10.1046/j.1523-1755.2002.00380.x 12028447

[B2] Bülow R. D. Boor P. (2019). Extracellular matrix in kidney fibrosis: more than just a scaffold. J. Histochem Cytochem 67, 643–661. 10.1369/0022155419849388 31116062 PMC6713975

[B3] Chen X. B. Yang Q. Y. Qin X. L. Zhao F. Y. Tang X. E. Wang Z. L. (2018). Effect of balanophora polysaccharide on expression of PPAH7, irisin and glucolipid metabolism in experimental diabetic rats. Chin. J. Pharmacol. Toxicol. 32 (5), 400–406. 10.3867/j.issn.1000-3002.2018.05.008

[B4] Chen J. Liu Q. He J. Li Y. (2022a). Immune responses in diabetic nephropathy: pathogenic mechanisms and therapeutic target. Front. Immunol. 13, 958790. 10.3389/fimmu.2022.958790 36045667 PMC9420855

[B5] Chen J. Tang Y. Zhong Y. Wei B. Huang X.-R. Tang P. M.-K. (2022b). P2Y12 inhibitor clopidogrel inhibits renal fibrosis by blocking macrophage-to-myofibroblast transition. Mol. Ther. 30, 3017–3033. 10.1016/j.ymthe.2022.06.019 35791881 PMC9481993

[B6] Chen Y. Chen R. Ji X. Zeng Z. Guan C. (2025). NLRP3 inflammasome-mediated pyroptosis in diabetic nephropathy: pathogenic mechanisms and therapeutic targets. J. Inflamm. Res. 18, 8399–8418. 10.2147/JIR.S524246 40585041 PMC12206412

[B7] Dalal D. Singh L. Singh A. (2025). Calycosin and kidney health: a molecular perspective on its protective mechanisms. Pharmacol. Rep. 77, 658–669. 10.1007/s43440-025-00728-3 40249500

[B8] Dwivedi S. Sikarwar M. S. (2025). Diabetic nephropathy: pathogenesis, mechanisms, and therapeutic strategies. Horm. Metab. Res. 57, 7–17. 10.1055/a-2435-8264 39572154

[B9] Eikmans M. Baelde J. J. de Heer E. Bruijn J. A. (2003). ECM homeostasis in renal diseases: a genomic approach. J. Pathol. 200, 526–536. 10.1002/path.1417 12845620

[B10] Fu Y. Wu N. Zhao D. (2017). Function of NLRP3 in the pathogenesis and development of diabetic nephropathy. Med. Sci. Monit. 23, 3878–3884. 10.12659/msm.903269 28798291 PMC5565226

[B11] Guo C. He Y. Gai L. Qu J. Shi Y. Xu W. (2020). Balanophora polyandra griff. Prevents dextran sulfate sodium-induced murine experimental colitis *via* the regulation of NF-κB and NLRP3 inflammasome. Food Funct. 11, 6104–6114. 10.1039/c9fo02494h 32572399

[B12] Guo Y. Zhao Y. Qiao Y. Xing Y. Fang Y. Zhao Y. (2025). Targeting panoptosis: a narrative review of its therapeutic potential in kidney disease. BMC Nephrol. 26, 545. 10.1186/s12882-025-04339-1 41023669 PMC12481750

[B13] Gupta A. Singh K. Fatima S. Ambreen S. Zimmermann S. Younis R. (2022). Neutrophil extracellular traps promote NLRP3 inflammasome activation and glomerular endothelial dysfunction in diabetic kidney disease. Nutrients 14, 2965. 10.3390/nu14142965 35889923 PMC9320009

[B14] Han Y. Xu X. Tang C. Gao P. Chen X. Xiong X. (2018). Reactive oxygen species promote tubular injury in diabetic nephropathy: the role of the mitochondrial ros-txnip-nlrp3 biological axis. Redox Biol. 16, 32–46. 10.1016/j.redox.2018.02.013 29475133 PMC5842313

[B15] Humphreys B. D. (2018). Mechanisms of renal fibrosis. Annu. Rev. Physiol. 80, 309–326. 10.1146/annurev-physiol-022516-034227 29068765

[B16] Jia W. Yu R. Wang L. Zhu D. Guo L. Weng J. (2025). Prevalence of chronic kidney disease among Chinese adults with diabetes: a nationwide population-based cross-sectional study. Lancet Reg. Health West Pac 55, 101463. 10.1016/j.lanwpc.2024.101463 39882253 PMC11773038

[B17] Jing Z. Hu L. Su Y. Ying G. Ma C. Wei J. (2021). Potential signaling pathway through which notch regulates oxidative damage and apoptosis in renal tubular epithelial cells induced by high glucose. J. Recept Signal Transduct. Res. 41, 357–362. 10.1080/10799893.2020.1810706 32933345

[B18] Jo E.-K. Kim J. K. Shin D.-M. Sasakawa C. (2016). Molecular mechanisms regulating NLRP3 inflammasome activation. Cell Mol. Immunol. 13, 148–159. 10.1038/cmi.2015.95 26549800 PMC4786634

[B19] Kanwar Y. S. Sun L. Xie P. Liu F.-Y. Chen S. (2011). A glimpse of various pathogenetic mechanisms of diabetic nephropathy. Annu. Rev. Pathol. 6, 395–423. 10.1146/annurev.pathol.4.110807.092150 21261520 PMC3700379

[B20] Kelley N. Jeltema D. Duan Y. He Y. (2019). The NLRP3 inflammasome: an overview of mechanisms of activation and regulation. Int. J. Mol. Sci. 20, 3328. 10.3390/ijms20133328 31284572 PMC6651423

[B21] Kim K. K. Sheppard D. Chapman H. A. (2018). TGF-β1 signaling and tissue fibrosis. Cold Spring Harb. Perspect. Biol. 10, a022293. 10.1101/cshperspect.a022293 28432134 PMC5880172

[B22] Lee S. C. Han S. H. Li J. J. Lee S. H. Jung D.-S. Kwak S.-J. (2009). Induction of heme oxygenase-1 protects against podocyte apoptosis under diabetic conditions. Kidney Int. 76, 838–848. 10.1038/ki.2009.286 19657327

[B23] Lee H. Fessler M. B. Qu P. Heymann J. Kopp J. B. (2020). Macrophage polarization in innate immune responses contributing to pathogenesis of chronic kidney disease. BMC Nephrol. 21, 270. 10.1186/s12882-020-01921-7 32660446 PMC7358194

[B24] Li Y. Teng D. Shi X. Qin G. Qin Y. Quan H. (2020). Prevalence of diabetes recorded in mainland China using 2018 diagnostic criteria from the American diabetes association: national cross sectional study. BMJ 369, m997. 10.1136/bmj.m997 32345662 PMC7186854

[B25] Li L. Zhou G. Fu R. He Y. Xiao L. Peng F. (2021). Polysaccharides extracted from balanophora polyandra griff (BPP) ameliorate renal fibrosis and EMT *via* inhibiting the hedgehog pathway. J. Cell Mol. Med. 25, 2828–2840. 10.1111/jcmm.16313 33507617 PMC7957266

[B26] Li J. Zhang J. Yang M. Huang X. Zhang M. Fang X. (2022). Kirenol alleviates diabetic nephropathy *via* regulating TGF-β/smads and the NF-κB signal pathway. Pharm. Biol. 60, 1690–1700. 10.1080/13880209.2022.2112239 36073930 PMC9467559

[B27] Liew A. Bavanandan S. Hao C.-M. Lim S. K. Prasad N. Sahay M. (2025). Executive summary of the asian pacific society of nephrology clinical practice guideline on diabetic kidney disease-2025 update. Nephrol. Carlt. 30, e70031. 10.1111/nep.70031 40325831 PMC12053225

[B28] Liu Y. Li Y. Xu L. Shi J. Yu X. Wang X. (2021). Quercetin attenuates podocyte apoptosis of diabetic nephropathy through targeting EGFR signaling. Front. Pharmacol. 12, 792777. 10.3389/fphar.2021.792777 35069207 PMC8766833

[B29] Ma R. C. W. (2018). Epidemiology of diabetes and diabetic complications in China. Diabetologia 61, 1249–1260. 10.1007/s00125-018-4557-7 29392352

[B30] Nogueira A. Pires M. J. Oliveira P. A. (2017). Pathophysiological mechanisms of renal fibrosis: a review of animal models and therapeutic strategies. Vivo 31, 1–22. 10.21873/invivo.11019 28064215 PMC5354133

[B31] Otto G. (2018). IL-1β switches on kidney fibrosis. Nat. Rev. Nephrol. 14, 475. 10.1038/s41581-018-0026-2 29799011

[B32] Qi J. Tongtong Q. Yuan L. Liu D. Yang L. Mao H. (2023). Oxidative stress and inflammation in diabetic nephropathy: role of polyphenols. Front. Immunol. 14, 1185317. 10.3389/fimmu.2023.1185317 37545494 PMC10401049

[B33] Qiu Y.-Y. Tang L.-Q. (2016). Roles of the NLRP3 inflammasome in the pathogenesis of diabetic nephropathy. Pharmacol. Res. 114, 251–264. 10.1016/j.phrs.2016.11.004 27826011

[B34] Qiu D. Song S. Chen N. Bian Y. Yuan C. Zhang W. (2023). NQO1 alleviates renal fibrosis by inhibiting the TLR4/NF-κB and TGF-β/smad signaling pathways in diabetic nephropathy. Cell Signal 108, 110712. 10.1016/j.cellsig.2023.110712 37196773

[B35] Rivero A. Mora C. Muros M. García J. Herrera H. Navarro-González J. F. (2009). Pathogenic perspectives for the role of inflammation in diabetic nephropathy. Clin. Sci. (Lond) 116, 479–492. 10.1042/CS20080394 19200057

[B36] Samsu N. (2021). Diabetic nephropathy: challenges in pathogenesis, diagnosis, and treatment. BioMed Res. Int. 2021, 1497449. 10.1155/2021/1497449 34307650 PMC8285185

[B37] Suriano F. Vieira-Silva S. Falony G. Roumain M. Paquot A. Pelicaen R. (2021). Novel insights into the genetically obese (ob/ob) and diabetic (db/db) mice: two sides of the same coin. Microbiome 9, 147. 10.1186/s40168-021-01097-8 34183063 PMC8240277

[B38] Venugopal H. Hanna A. Humeres C. Frangogiannis N. G. (2022). Properties and functions of fibroblasts and myofibroblasts in myocardial infarction. Cells 11, 1386. 10.3390/cells11091386 35563692 PMC9102016

[B39] Wada J. Makino H. (2013). Inflammation and the pathogenesis of diabetic nephropathy. Clin. Sci. (Lond) 124, 139–152. 10.1042/CS20120198 23075333

[B40] Wan J. Liu D. Pan S. Zhou S. Liu Z. (2022). NLRP3-mediated pyroptosis in diabetic nephropathy. Front. Pharmacol. 13, 998574. 10.3389/fphar.2022.998574 36304156 PMC9593054

[B41] Wang Y. Zhang X. Mao Y. Liang L. Liu L. Peng W. (2020). Smad2 and Smad3 play antagonistic roles in high glucose-induced renal tubular fibrosis *via* the regulation of SnoN. Exp. Mol. Pathol. 113, 104375. 10.1016/j.yexmp.2020.104375 31917288

[B42] Wang F. Liu C. Ren L. Li Y. Yang H. Yu Y. (2023). Sanziguben polysaccharides improve diabetic nephropathy in mice by regulating gut microbiota to inhibit the TLR4/NF-κB/NLRP3 signalling pathway. Pharm. Biol. 61, 427–436. 10.1080/13880209.2023.2174145 36772833 PMC9930838

[B43] Wu M. Han W. Song S. Du Y. Liu C. Chen N. (2018). NLRP3 deficiency ameliorates renal inflammation and fibrosis in diabetic mice. Mol. Cell Endocrinol. 478, 115–125. 10.1016/j.mce.2018.08.002 30098377

[B44] Xiang E. Han B. Zhang Q. Rao W. Wang Z. Chang C. (2020). Human umbilical cord-derived mesenchymal stem cells prevent the progression of early diabetic nephropathy through inhibiting inflammation and fibrosis. Stem Cell Res. Ther. 11, 336. 10.1186/s13287-020-01852-y 32746936 PMC7397631

[B45] Xu G. Yuan H. Liu J. Wang X. Ma L. Wang Y. (2025). Astragalus mongholicus polysaccharides alleviate kidney injury in rats with type 2 diabetes through modulation of oxidation, inflammation, and gut microbiota. Int. J. Mol. Sci. 26, 1470. 10.3390/ijms26041470 40003935 PMC11855448

[B46] Yang M. Zhang C. (2024). The role of innate immunity in diabetic nephropathy and their therapeutic consequences. J. Pharm. Anal. 14, 39–51. 10.1016/j.jpha.2023.09.003 38352948 PMC10859537

[B47] Yang M. Zhao L. (2023). The selective NLRP3-inflammasome inhibitor CY-09 ameliorates kidney injury in diabetic nephropathy by inhibiting NLRP3- inflammasome activation. Curr. Med. Chem. 30, 3261–3270. 10.2174/0929867329666220922104654 36154582

[B48] Yosri H. El-Kashef D. H. El-Sherbiny M. Said E. Salem H. A. (2022). Calycosin modulates NLRP3 and TXNIP-mediated pyroptotic signaling and attenuates diabetic nephropathy progression in diabetic rats; an insight. Biomed. Pharmacother. 155, 113758. 10.1016/j.biopha.2022.113758 36271546

[B49] Zanchi C. Macconi D. Trionfini P. Tomasoni S. Rottoli D. Locatelli M. (2017). MicroRNA-184 is a downstream effector of albuminuria driving renal fibrosis in rats with diabetic nephropathy. Diabetologia 60, 1114–1125. 10.1007/s00125-017-4248-9 28364255 PMC5423990

[B50] Zhao X. Kwan J. Y. Y. Yip K. Liu P. P. Liu F.-F. (2020). Targeting metabolic dysregulation for fibrosis therapy. Nat. Rev. Drug Discov. 19, 57–75. 10.1038/s41573-019-0040-5 31548636

[B51] Zheng Z. Ma T. Lian X. Gao J. Wang W. Weng W. (2019). Clopidogrel reduces fibronectin accumulation and improves diabetes-induced renal fibrosis. Int. J. Biol. Sci. 15, 239–252. 10.7150/ijbs.29063 30662363 PMC6329922

