# Supplementary material for: Balanophora polysaccharide improves renal injury and fibrosis in db/db diabetic nephropathy mice via NLRP3 inflammasome mediated inflammation
Source: Front Pharmacol. 2025 Nov 28;16:1671678. doi: 10.3389/fphar.2025.1671678 (PMC12698541; doi:10.3389/fphar.2025.1671678)
Supplement: Supplementary file 1 [file DataSheet2.pdf]

ASC

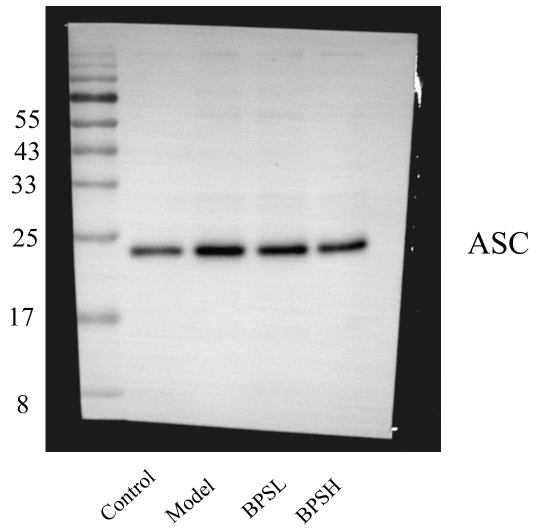

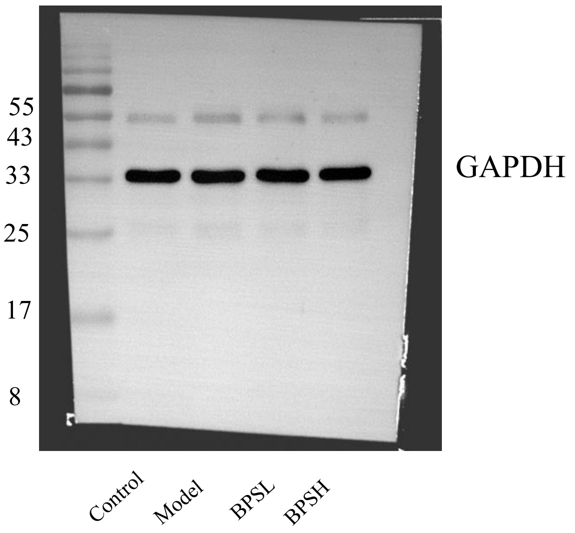

α-SMA

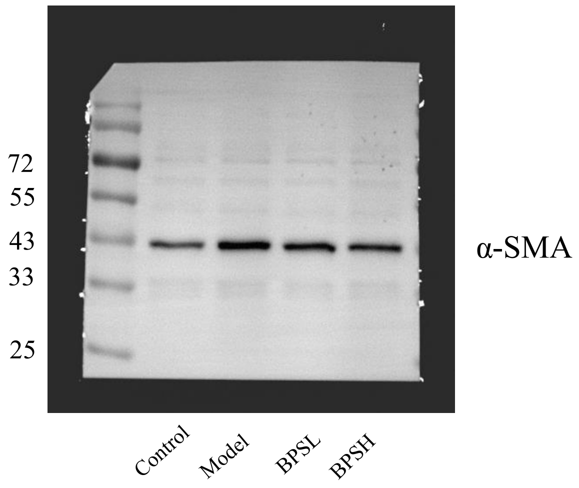

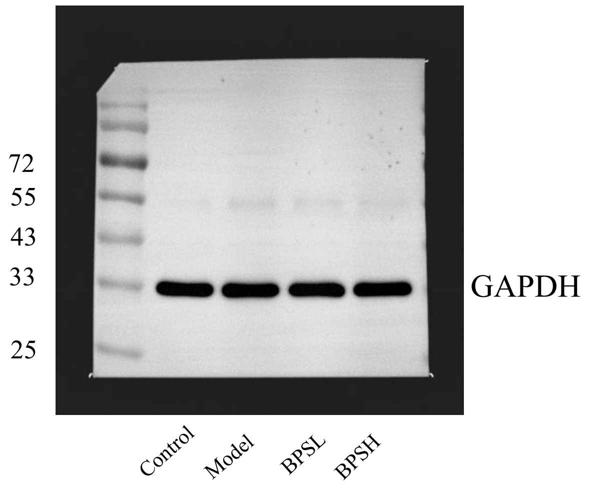

IL-1β

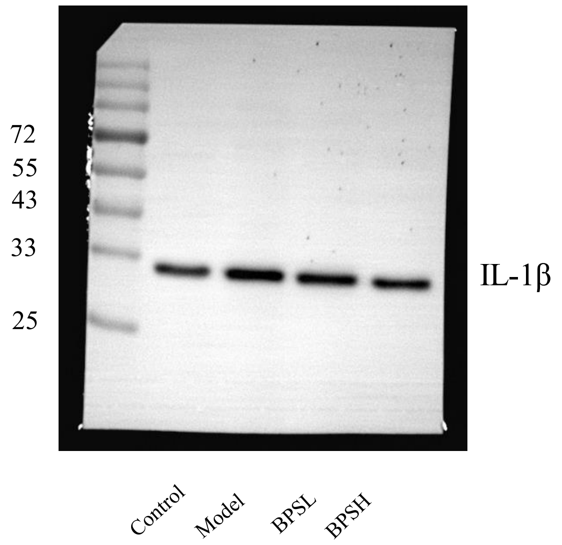

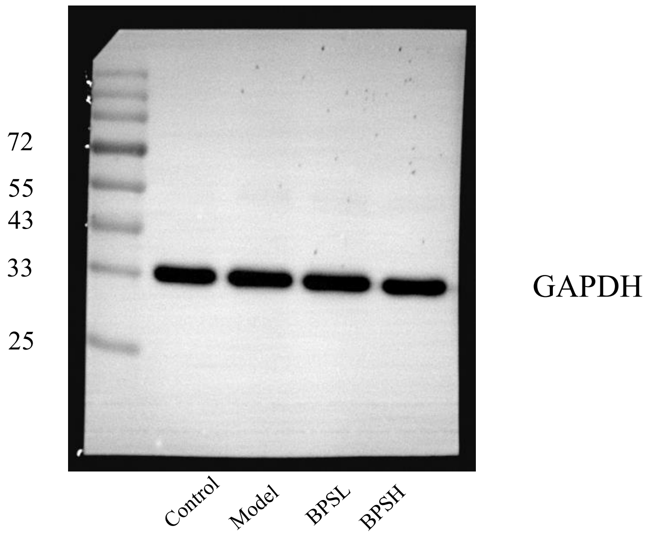

cleaved-Caspase1

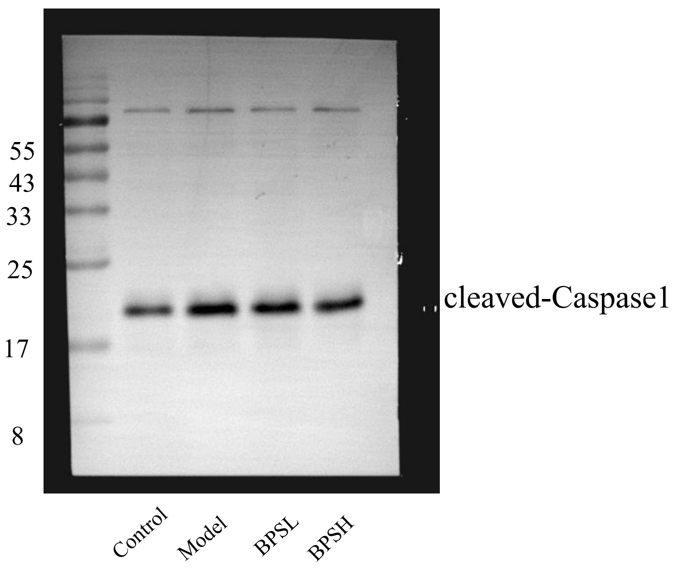

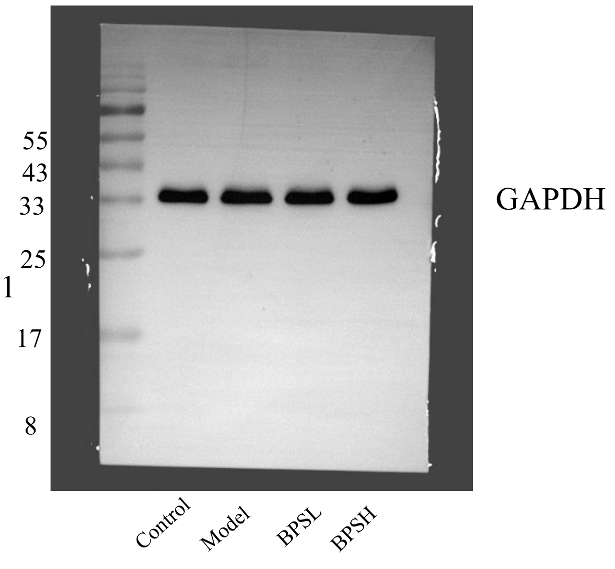

BAX

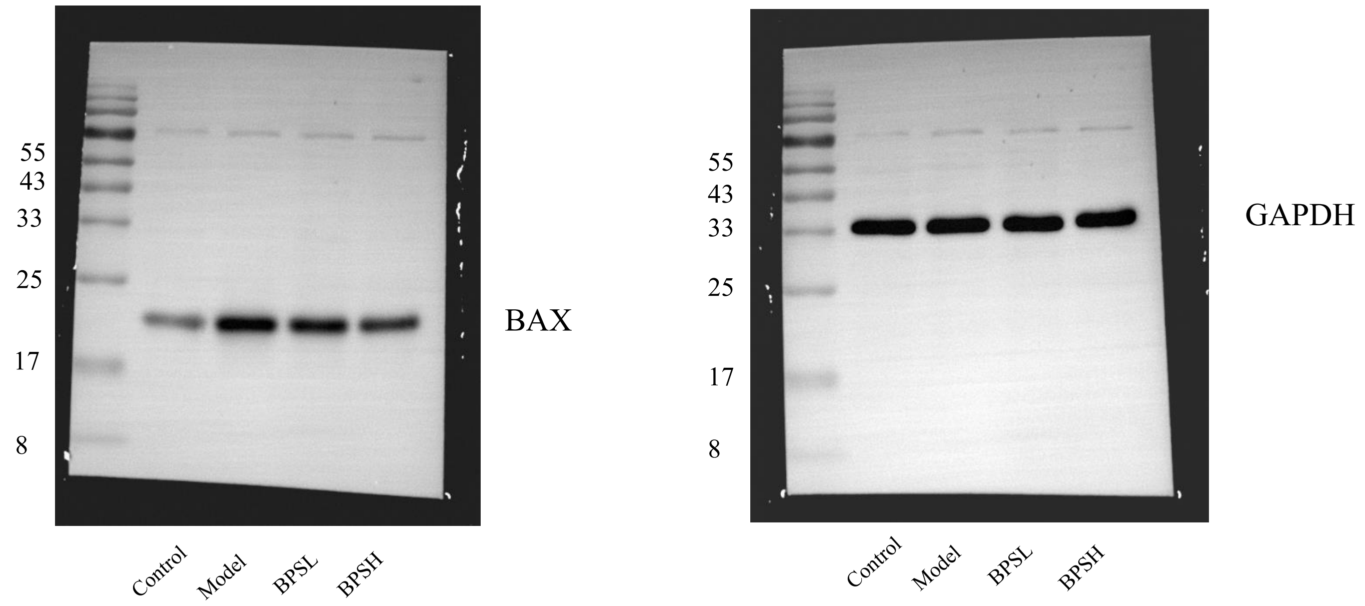

NF-κB

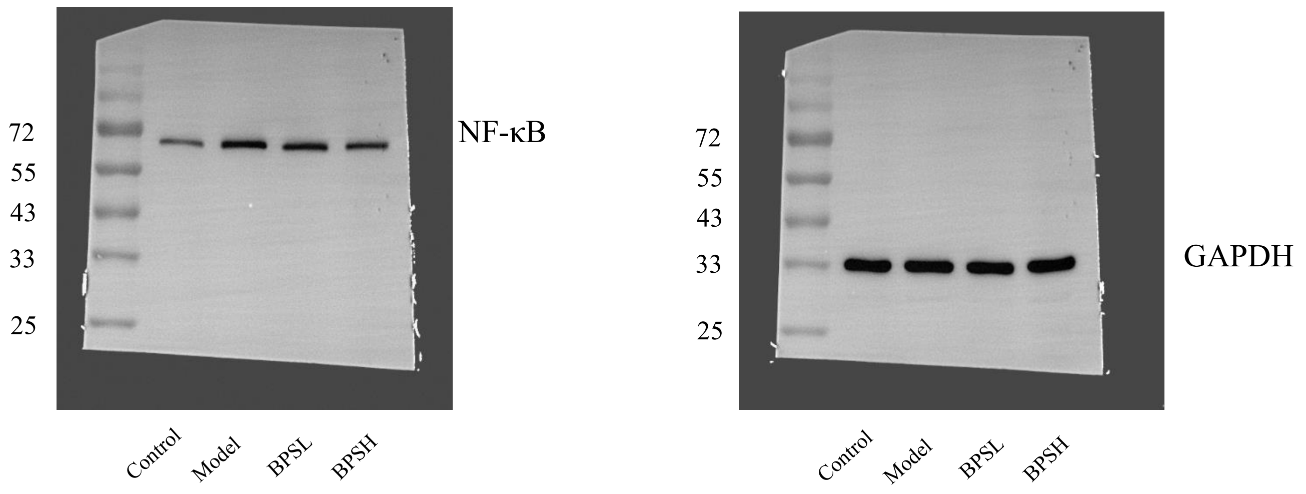

TNF-α

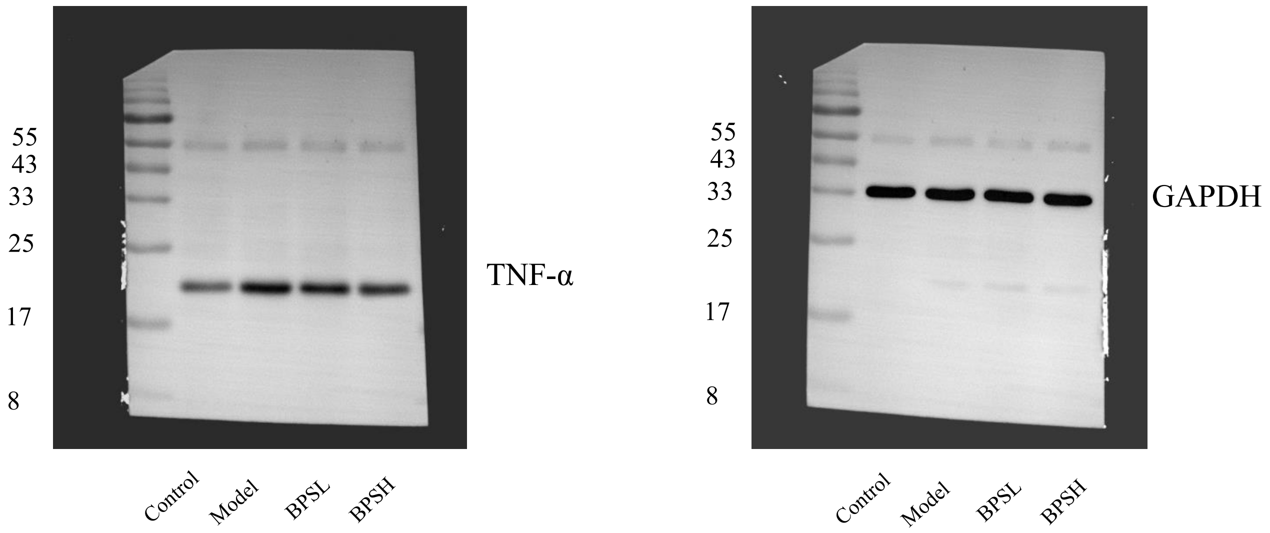

IL-10

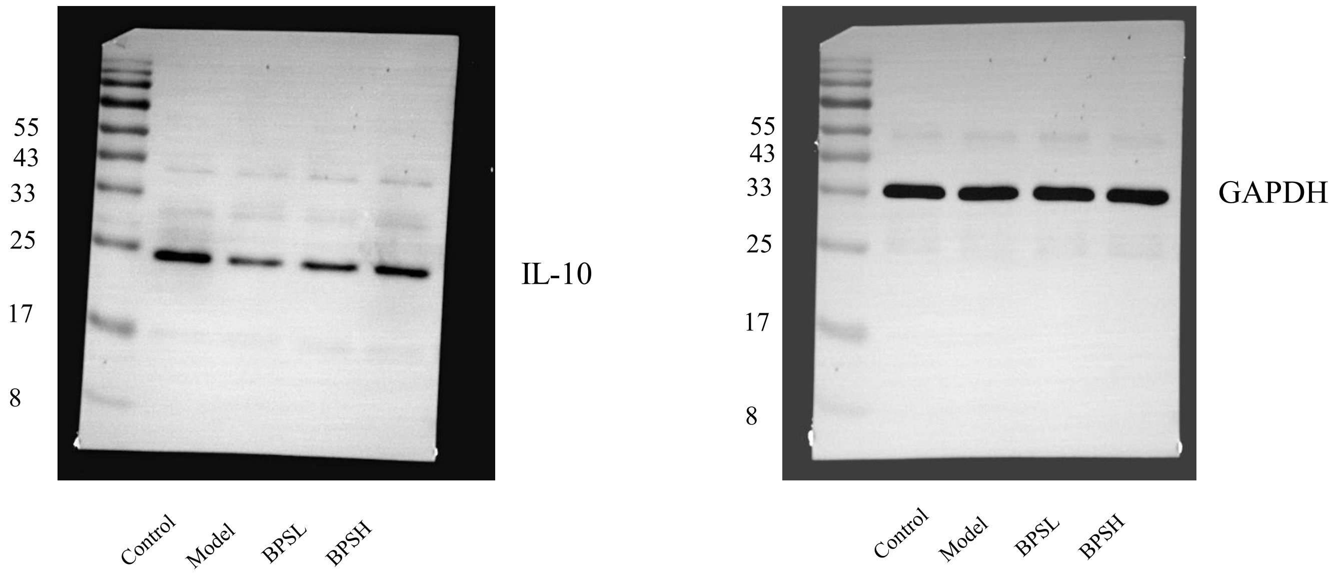

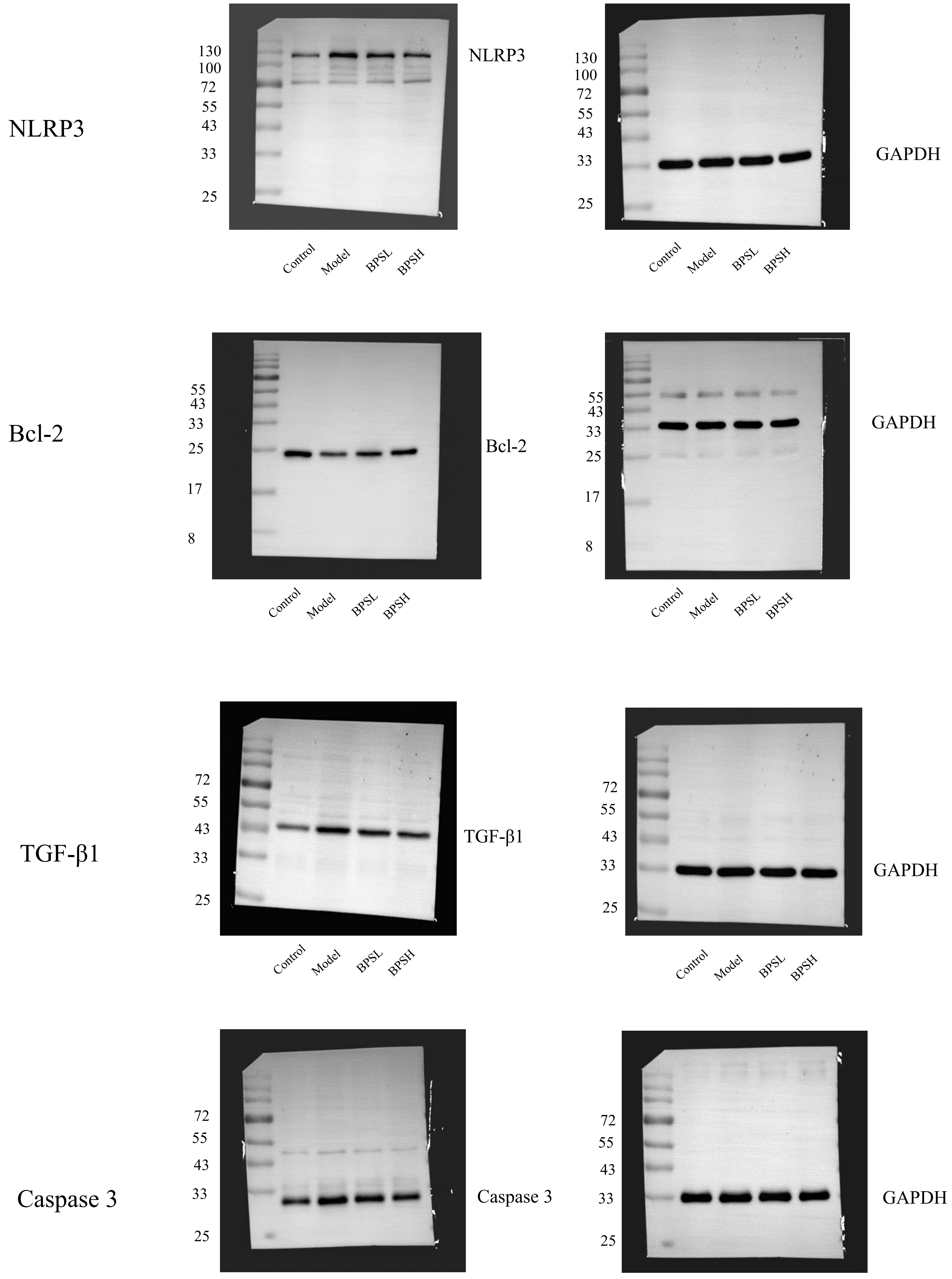
NLRP3
130
100
72
55
43
33
25
NLRP3
GAPDH
Control
Model
BPSL
BPSH
Bcl-2
55
43
33
25
17
8
Bcl-2
GAPDH
TGF-β1
72
55
43
33
25
TGF-β1
GAPDH
Caspase 3
Caspase 3
GAPDH
