# Supplementary material for: Balanophora polysaccharide improves renal injury and fibrosis in db/db diabetic nephropathy mice via NLRP3 inflammasome mediated inflammation
Source: Front Pharmacol. 2025 Nov 28;16:1671678. doi: 10.3389/fphar.2025.1671678 (PMC12698541; doi:10.3389/fphar.2025.1671678)
Supplement: Supplementary file 2 [file DataSheet4.pdf]

ESI+

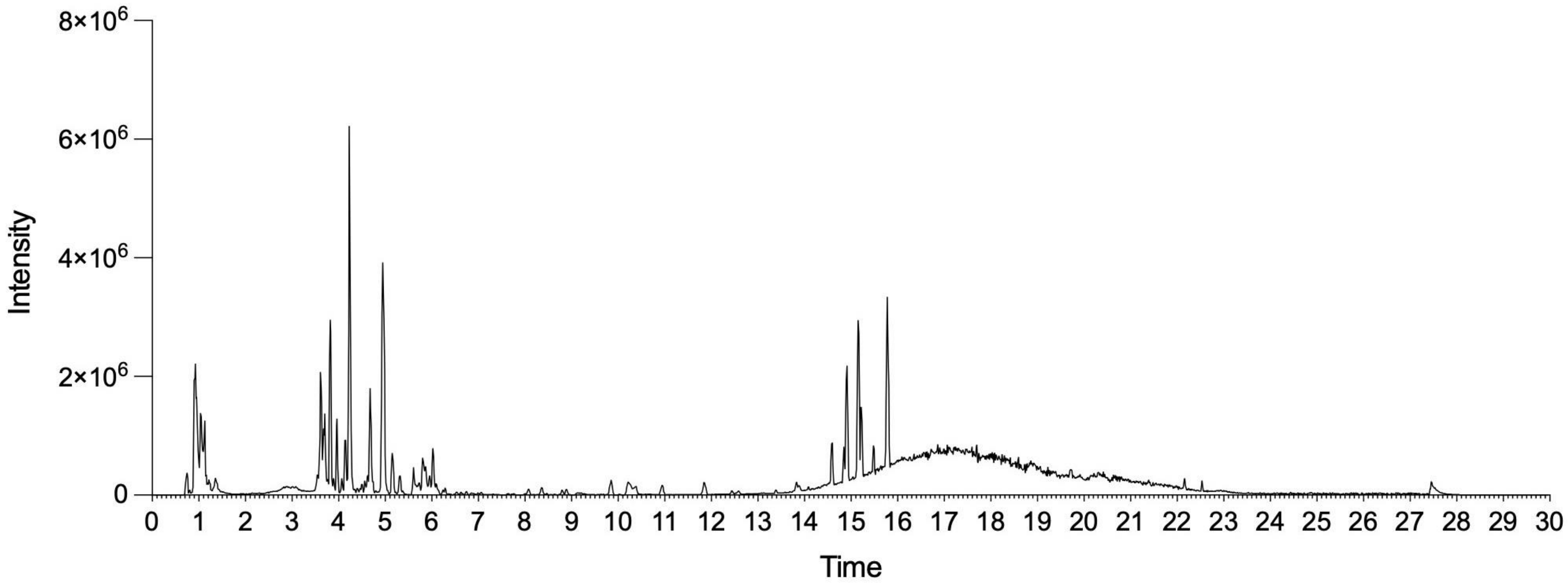

ESI-

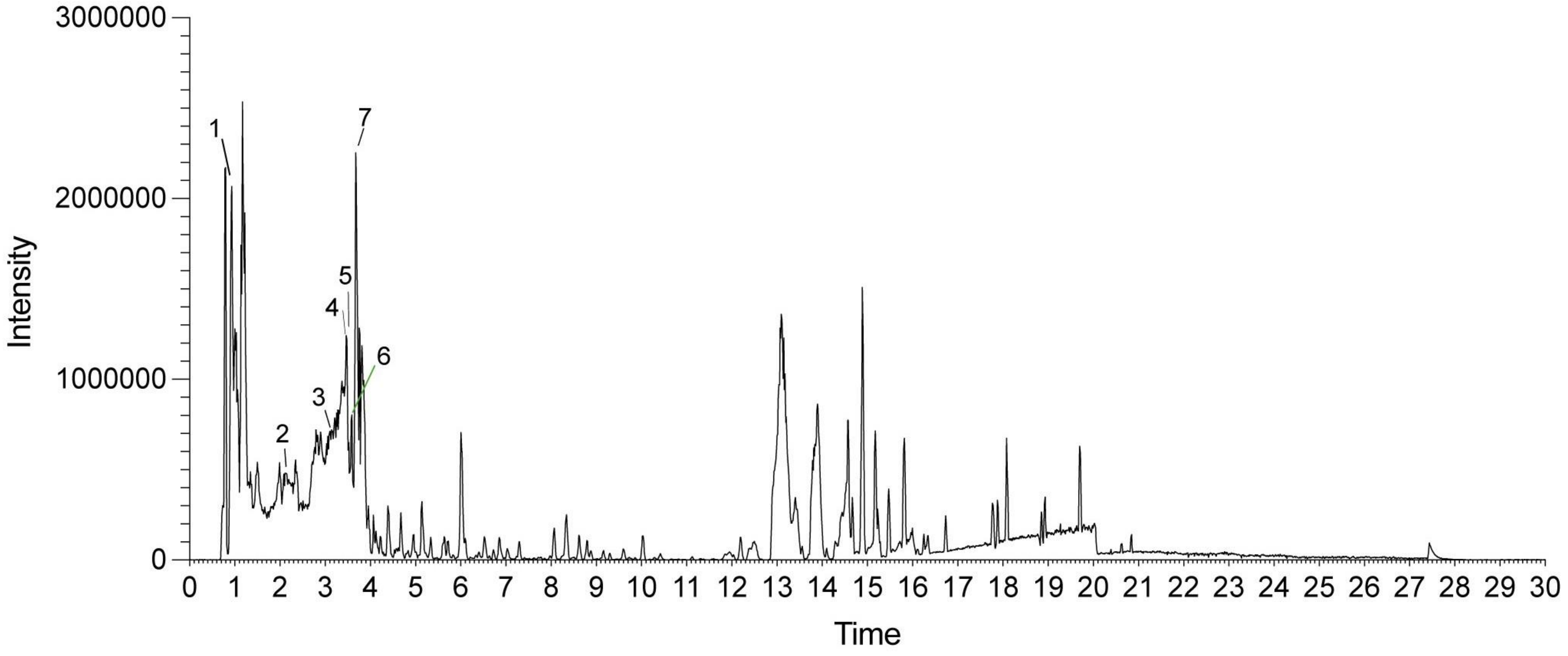

Table 1 Identification of Carbohydrate Compounds in Balanophora involucrata

	Compounds	Formula	Delta (PPM)	RT (MIN)	Lonization mode	m/z
1	D-Glucose	$C_6H_{12}O_6$	1.6196	0.914	[M-H]-	179.05612
2	6-O-galloyl-beta-D-glucose	$C_{13}H_{16}O_{10}$	1.44986	2.138	[M-H]-	331.06689
3	1-O-Galloyl-beta-D-glucose	$C_{13}H_{16}O_{10}$	2.02376	3.102	[M-H]-	331.06741
4	1-Caffeoyl-beta-D-glucose	$C_{15}H_{18}O_9$	9.20583	3.519	[M-H]-	341.0881
5	1-O,6-O-Digalloyl-beta-D-glucose	$C_{20}H_{20}O_{14}$	3.5398	3.519	[M-H]-	483.0777
6	1,2,3-Tri-O-galloyl-beta-D-glucose	$C_{27}H_{24}O_{18}$	2.97596	3.552	[M-H]-	635.08905
7	1,2,3,6-Tetragalloylglucose	$C_{34}H_{28}O_{22}$	2.55368	3.643	[M-H]-	787.09991
