# Supplementary material for: Balanophora polysaccharide improves renal injury and fibrosis in db/db diabetic nephropathy mice via NLRP3 inflammasome mediated inflammation
Source: Front Pharmacol. 2025 Nov 28;16:1671678. doi: 10.3389/fphar.2025.1671678 (PMC12698541; doi:10.3389/fphar.2025.1671678)

D-Glucose

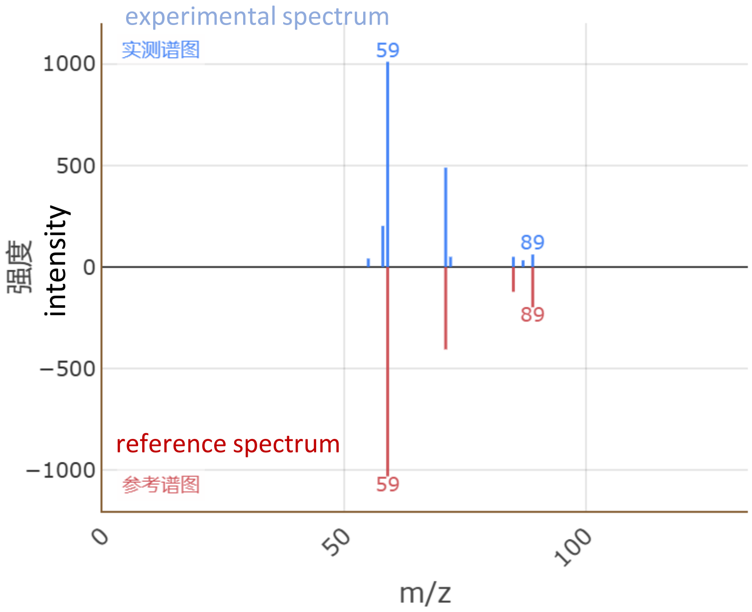

6-O-galloyl-beta-D-glucose

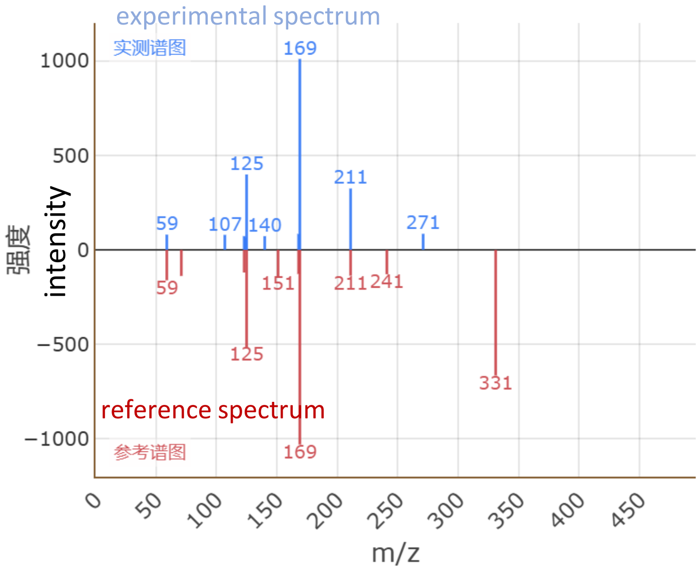

1-O-Galloyl-beta-D-glucose

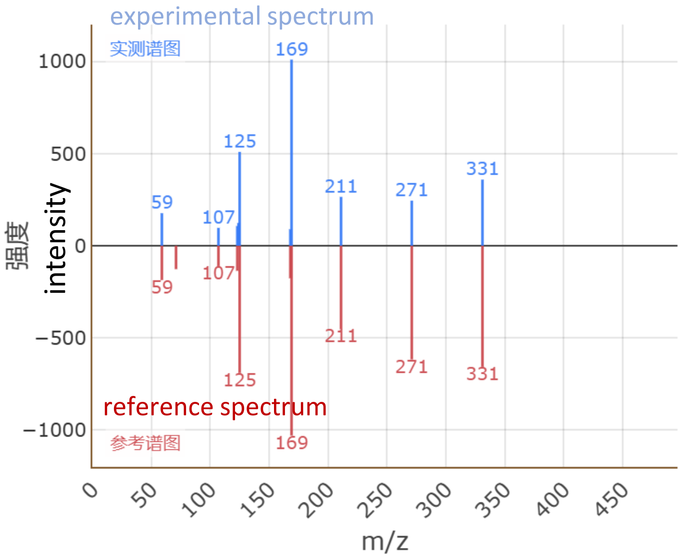

1-O,6-O-Digalloyl-beta-D-glucose1

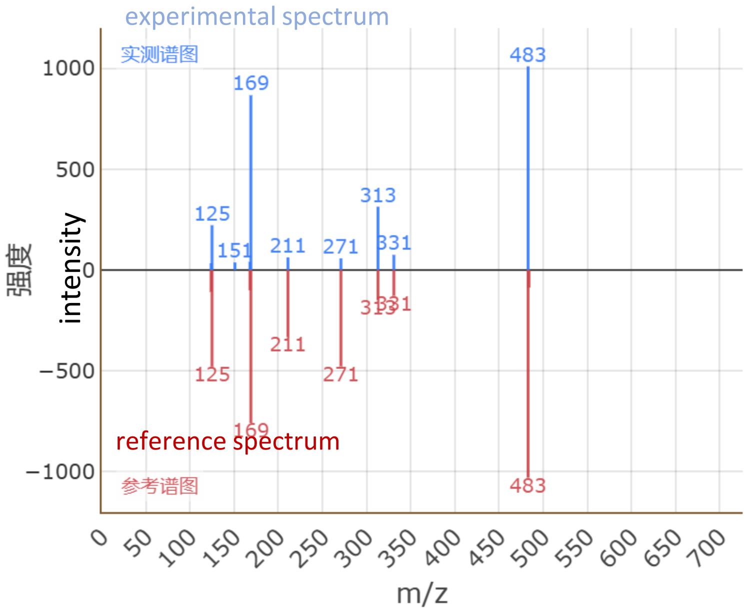

1,2,3,6-Tetragalloylglucose1

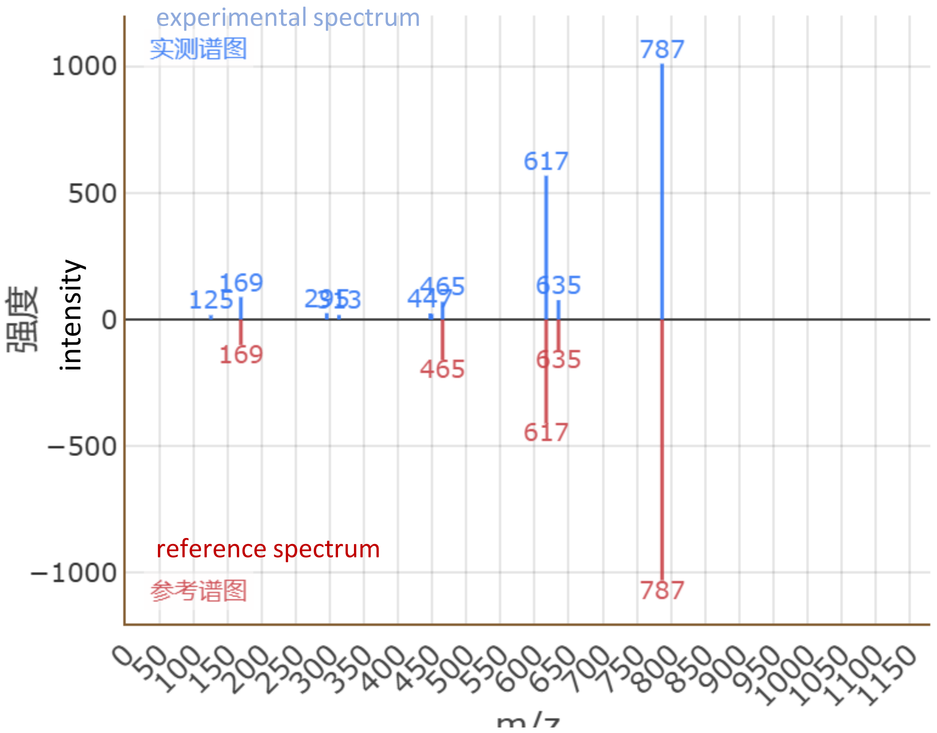

1,2,3-Tri-O-galloyl-beta-D-glucose

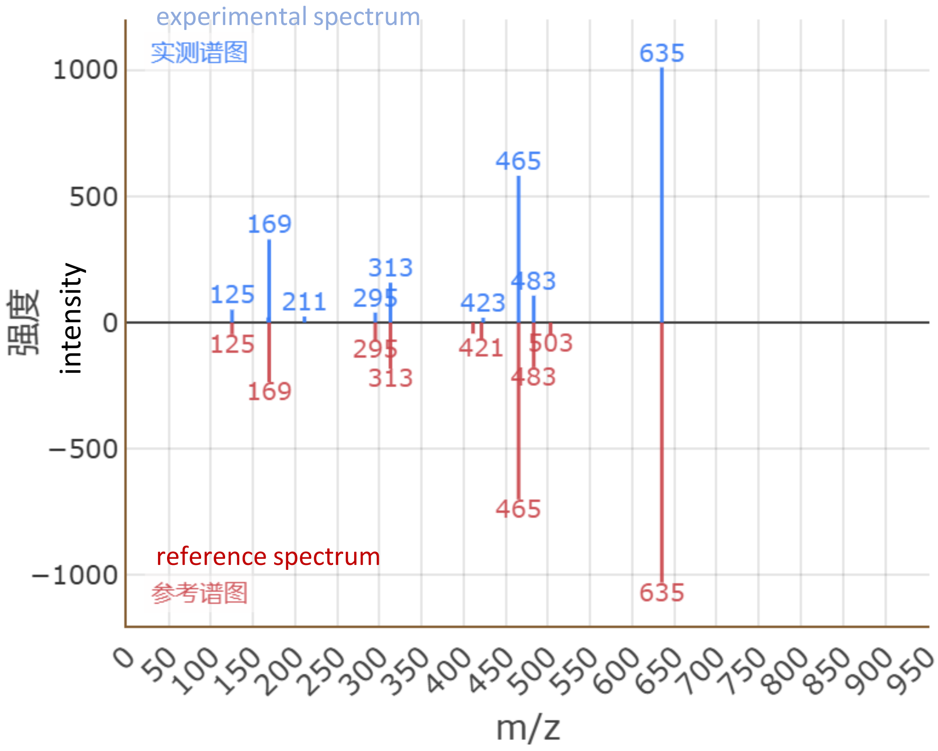

1-Caffeoyl-beta-D-glucose

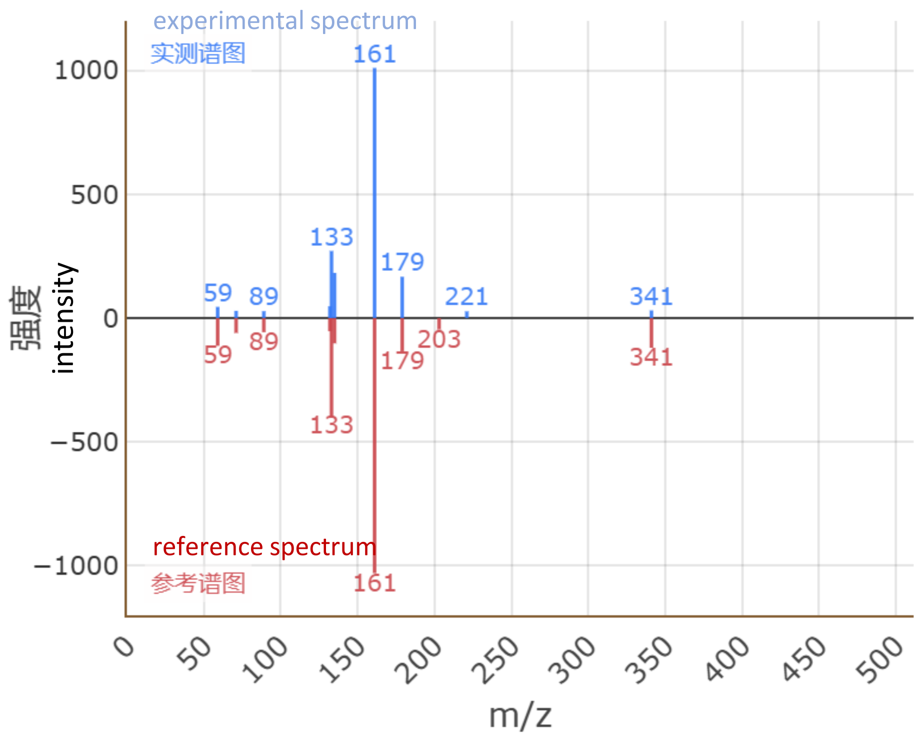

Supplement: Supplementary file 3 [file DataSheet3.pdf]
