# Supplementary material for: Balanophora polysaccharide improves renal injury and fibrosis in db/db diabetic nephropathy mice via NLRP3 inflammasome mediated inflammation
Source: Front Pharmacol. 2025 Nov 28;16:1671678. doi: 10.3389/fphar.2025.1671678 (PMC12698541; doi:10.3389/fphar.2025.1671678)
Supplement: Supplementary file 4 [file DataSheet1.pdf]

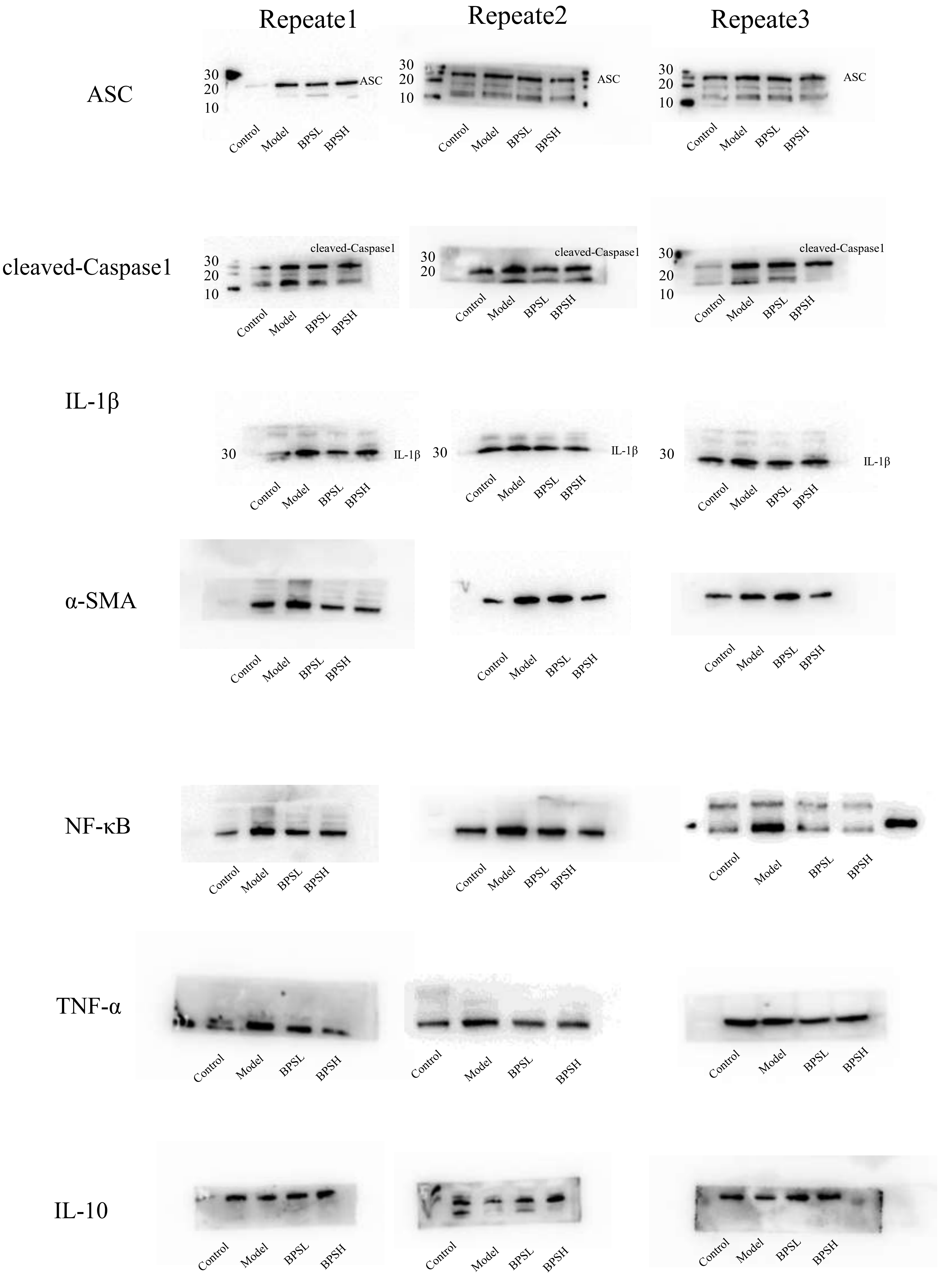

Repeate1
Repeate2
Repeate3
ASC
cleaved-Caspase1
IL-1β
α-SMA
NF-κB
TNF-α
IL-10
30
20
10
Control
Model
BPSL
BPSH

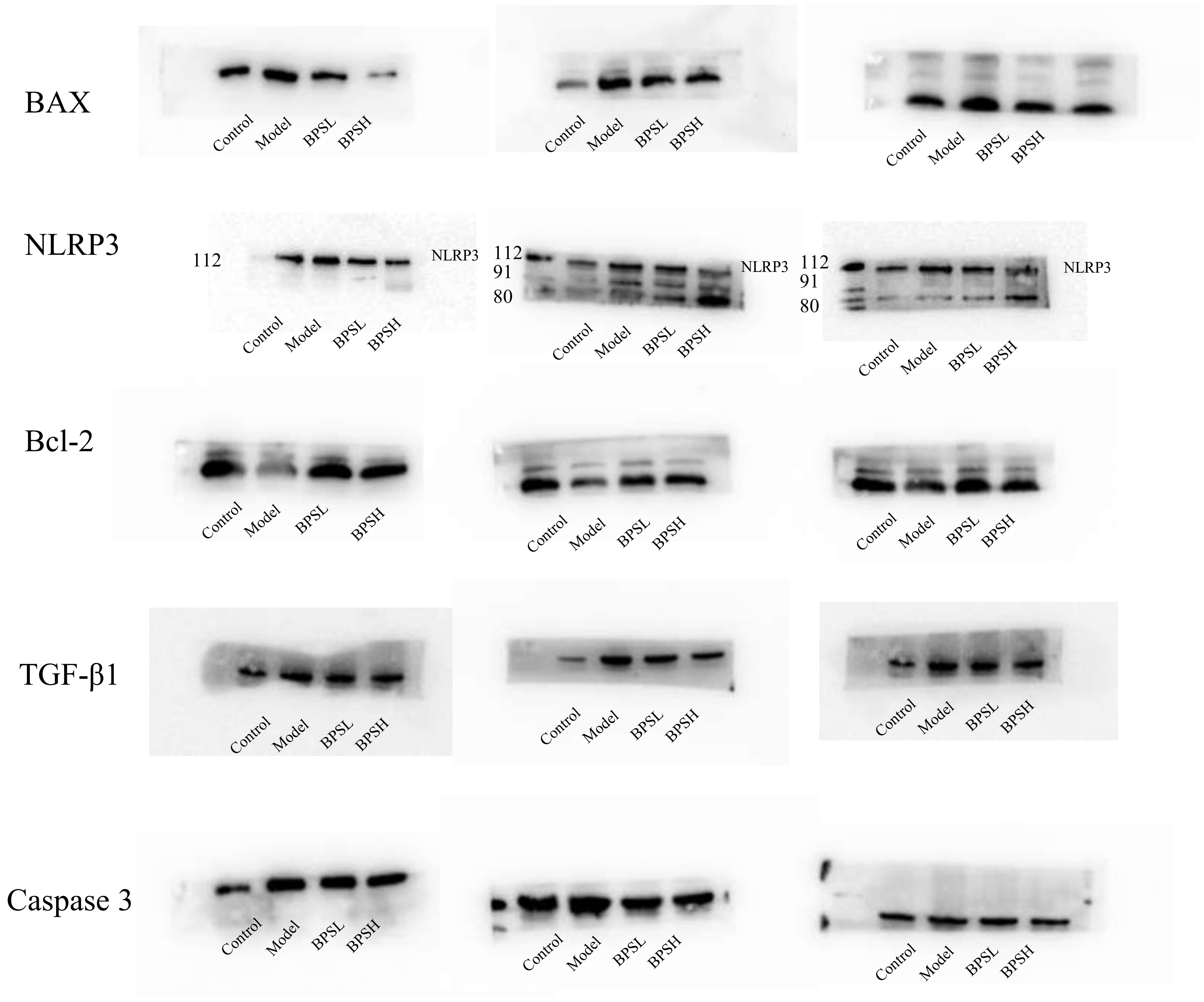
BAX
NLRP3
Bcl-2
TGF-β1
Caspase 3
Control
Model
BPSL
BPSH
112
91
80
NLRP3
